# Close-kin mark-recapture informs critically endangered terrestrial mammal status

**DOI:** 10.1038/s41598-023-38639-z

**Published:** 2023-08-02

**Authors:** Luke R. Lloyd-Jones, Mark V. Bravington, Kyle N. Armstrong, Emma Lawrence, Pierre Feutry, Christopher M. Todd, Annabel Dorrestein, Justin A. Welbergen, John M. Martin, Karrie Rose, Jane Hall, David N. Phalen, Isabel Peters, Shane M. Baylis, Nicholas A. Macgregor, David A. Westcott

**Affiliations:** 1 Commonwealth Scientific and Industrial Research Organisation, Data61, Brisbane, QLD 4072 Australia; 2 Commonwealth Scientific and Industrial Research Organisation, Data61, Hobart, TAS 7000 Australia; 3 Environment Institute, University of Adelaide, North Terrace, Adelaide, South Australia 5005 Australia; 4 Commonwealth Scientific and Industrial Research Organisation, Oceans and Atmosphere, Hobart, TAS 7000 Australia; 5 The Hawkesbury Institute for the Environment, Western Sydney University, Richmond, NSW Australia; 6 Royal Botanic Gardens and Domain Trust, Sydney, NSW 2000 Australia; 7 Australian Registry of Wildlife Health, Taronga Conservation Society Australia, Bradleys Head Road, Mosman, NSW 2088 Australia; 8 Sydney School of Veterinary Science, Faculty of Science, University of Sydney, Sydney, NSW 2006 Australia; 9 School of Mathematics and Physics, University of Queensland, Brisbane, QLD 4072 Australia; 10 Parks Australia, Canberra, ACT 2601 Australia; 11 Durrell Institute of Conservation and Ecology (DICE), School of Anthropology and Conservation, University of Kent, Canterbury, CT2 7NR Kent UK; 12 Land and Water, Commonwealth Scientific and Industrial Research Organisation, Atherton, QLD 4883 Australia

**Keywords:** Ecology, Evolution, Genetics, Ecology, Environmental sciences, Mathematics and computing

## Abstract

Reliable information on population size is fundamental to the management of threatened species. For wild species, mark-recapture methods are a cornerstone of abundance estimation. Here, we show the first application of the close-kin mark-recapture (CKMR) method to a terrestrial species of high conservation value; the Christmas Island flying-fox (CIFF). The CIFF is the island's last remaining native terrestrial mammal and was recently listed as critically endangered. CKMR is a powerful tool for estimating the demographic parameters central to CIFF management and circumvents the complications arising from the species’ cryptic nature, mobility, and difficult-to-survey habitat. To this end, we used genetic data from 450 CIFFs captured between 2015 and 2019 to detect kin pairs. We implemented a novel CKMR model that estimates sex-specific abundance, trend, and mortality and accommodates observations from the kin-pair distribution of male reproductive skew and mate persistence. CKMR estimated CIFF total adult female abundance to be approximately 2050 individuals (95% CI (950, 4300)). We showed that on average only 23% of the adult male population contributed to annual reproduction and strong evidence for between-year mate fidelity, an observation not previously quantified for a *Pteropus* species in the wild. Critically, our population estimates provide the most robust understanding of the status of this critically endangered population, informing immediate and future conservation initiatives.

## Introduction

Reliable demographic information, and in particular knowledge of population size and trajectory, is fundamental to the effective management of species, whether they be exploited, controlled, or threatened^1,2^. Demographic data are essential to answering important questions in ecology and conservation concerning, for example, extinction prevention, harvest management, competition, predator–prey interactions, and emigration/immigration^3–6^. Estimating the population size of many wild species remains challenging because populations are typically large and dispersed, and individuals are often highly mobile and difficult to observe. Typically, mark-recapture experiments and methods are used to estimate population size and demographic parameters, with the foundations of the method going back over half a century^7,8^. With recent rapid development in genetic marker technology, genetic population size estimates have become feasible using analogues of mark-recapture studies in which individuals are sampled repeatedly and identified genetically^9^. More recent advances do not require repeated sampling but use genetic kinship as a replacement for recapture^10,11^. Genetic mark-recapture is yet to be deployed widely for threatened terrestrial species but advantages such as smaller required sample sizes, expected lower ongoing cost and high reliability make it a powerful alternative to traditional methods^12^.

The Christmas Island flying-fox (CIFF) (*Pteropus natalis*) is endemic to Christmas Island and is the island’s last remaining native terrestrial mammal^13,14^. Recent evidence suggests that the CIFF population is in decline^13,15,16^ with hypothesised drivers of this decline including chronic cadmium exposure, lack of food availability, disease, and interactions with yellow crazy ants, giant centipedes, and feral cats^13,17–19^. Surveys using distance sampling theory estimated the CIFF abundance to be approximately 2,100 individuals (standard error (SE) = 563)^17^, a substantial decline from the 1984 estimate of 6,000 individuals^20^. These and other factors prompted the listing of the CIFF under Australia’s federal Environment Protection and Biodiversity Conservation Act (1999) as critically endangered^21^. A subsequent capture-mark-recapture (abbreviated MR to facilitate differentiation from CKMR) study (Todd et al. 2020) estimated the population size to be 3846 (standard error (SE) interval 3643–4071) individuals in 2018, again well below Tidemann’s (1985) estimate. Population trends suggest that failure to address the causes of decline will likely lead to the species’ extinction, with Geyle et al.^22^ estimating that the CIFF has a 41% chance of extinction in the next 20 years. The decline of the CIFF, which is paralleled by that of other fauna on the island (including the extinction of the island’s only other bat species, *Pipistrellus murrayi* in 2009), has serious implications for the health of Christmas Island’s ecosystem. The CIFF is a keystone species that plays an important role in seed dispersal and pollination processes on the island^15,23,24^. Definitive demographic estimates for the CIFF population are essential to the management of this species but are difficult to obtain because of the CIFF’s cryptic nature, high mobility, the difficulty in accessing parts of the island, and incomplete knowledge of the species’ habitat use^16,23,24^.

The close-kin mark-recapture (CKMR) method^10,25^ can provide estimates of total adult abundance, rate of change in abundance, and survival, and effectively circumvents many of the traditional challenges of adequately surveying CIFFs. CKMR uses genotyping technology to recover a genetic *tag*, in the form of inherited genes, of an individual’s closely related kin, increasing the effective sample sizes relative to MR, which can only recover tagged individuals. CKMR generalises the application space of MR and offers great potential for populations that are difficult to survey and have low recapture rates, such as the CIFF population. CKMR is less prone to trap-happy/shy bias because individuals need only be captured once^26–28^. Furthermore, assumptions of sample mixing, mating system and reproductive biology can be readily checked via assessing the observed distribution of kin against their expectation under the assumed model. CKMR allows for inference on adults from the sampling of only juveniles^29^, and age-specific fecundities can be included^30^. Injuries associated with tagging and the potential changes to survival that may result from tags are also circumvented. Importantly, for harvested or hunted species, samples can be collected opportunistically from dead animals. To date, CKMR has been successfully applied to aquatic species only, including southern bluefin tuna^31^, salmon^32^, white, grey nurse, and northern river shark^11,33,34^, and validated against classical MR for brook trout^35^. There is considerable potential for the application of CKMR to terrestrial species with most species having the necessary reproductive biology for the method to be applicable (see Sect. 6. of Bravington et al.^10^ for examples of species where CKMR will not work).

In this study, we extended the CKMR principles to estimate the population ecology of the CIFF. We used genetic data from 450 CIFF individuals to investigate the distribution of kin pairs in the CIFF sample through pair-wise comparison of genetic data generated from the DArTseq genotyping technology^36–38^. Given the distribution of observed kin pairs, and their associated covariate information, we proposed a set of novel CKMR models, which included a female-focussed model and extended sex-specific models. We develop the CKMR theory to allow for the quantification of male reproductive skew i.e., the proportion of adult males that breed on average over breeding seasons, and the probability that male and female partners recouple year-on-year (referred to as mate persistence). We further applied mitochondrial DNA (mtDNA) sequencing techniques to inform the sex of the shared parent for the sex-specific analyses and incorporate the uncertainty in mtDNA haplotype sharing in the kinship probabilities and estimation procedure. We explored the adequacy of the CKMR models for providing demographic estimates for CIFF and quantified components of the CIFF mating system, which are essential for effective management. In this paper, we discuss the implications of the first application of CKMR to a terrestrial species and comment on its utility for monitoring CIFF and threatened populations more broadly.

## Results

### Sampling and tissue collection

Christmas Island is a territory of Australia located 1500 km west of the Australian mainland. Samples and measurements from individual CIFF were collected across the island during multiple sampling exercises for several different studies^19,24,39,40^ (see “Methods” for a detailed description). Wing-membrane biopsies for each individual were taken and stored in ethanol for genetic analyses. Across all studies, a total of 728 CIFF samples (≈ 38% female see Supplementary Table S1) with both tissue and phenotypic measurements were collected.

### Genetic detection of kin pairs

For CKMR, we are primarily interested in the genetic detection of parent–offspring pairs (POPs) and half-sibling pairs (HSPs). Wing-membrane biopsies were assessed with 693 of the 728 sampled individuals having available and substantial tissue for genetic analyses. DNA extraction and quality control were performed by Diversity Arrays Technology (DArT P/L, Canberra), with 197 individuals removed due to poor extraction and complexity reduction preparation, which was hypothesised to be a result of either tissue quality, microbial contamination, or both. To genetically infer kin pairs, we used the Kinference R package^41^ as the primary quality control and analytical tool. Following further quality control on genetic parameters across individuals and single-nucleotide polymorphisms (SNPs), 409 individuals and 2085 SNPs were retained for kinship analysis (see Supplementary Note A for detailed tissue preparation, genotyping and quality control procedures).

### First-degree relatives

To estimate kinship, the probability of the two observed genotypes at each locus for a pair of individuals was computed under the HSP versus unrelated pair kinship types. The likelihood ratio was then computed from these two probabilities and the sum of log-ratios taken as the overall statistic for the pair and is referred to as the pseudo-log-odds-ratio (PLOD).

**Figure 1:**
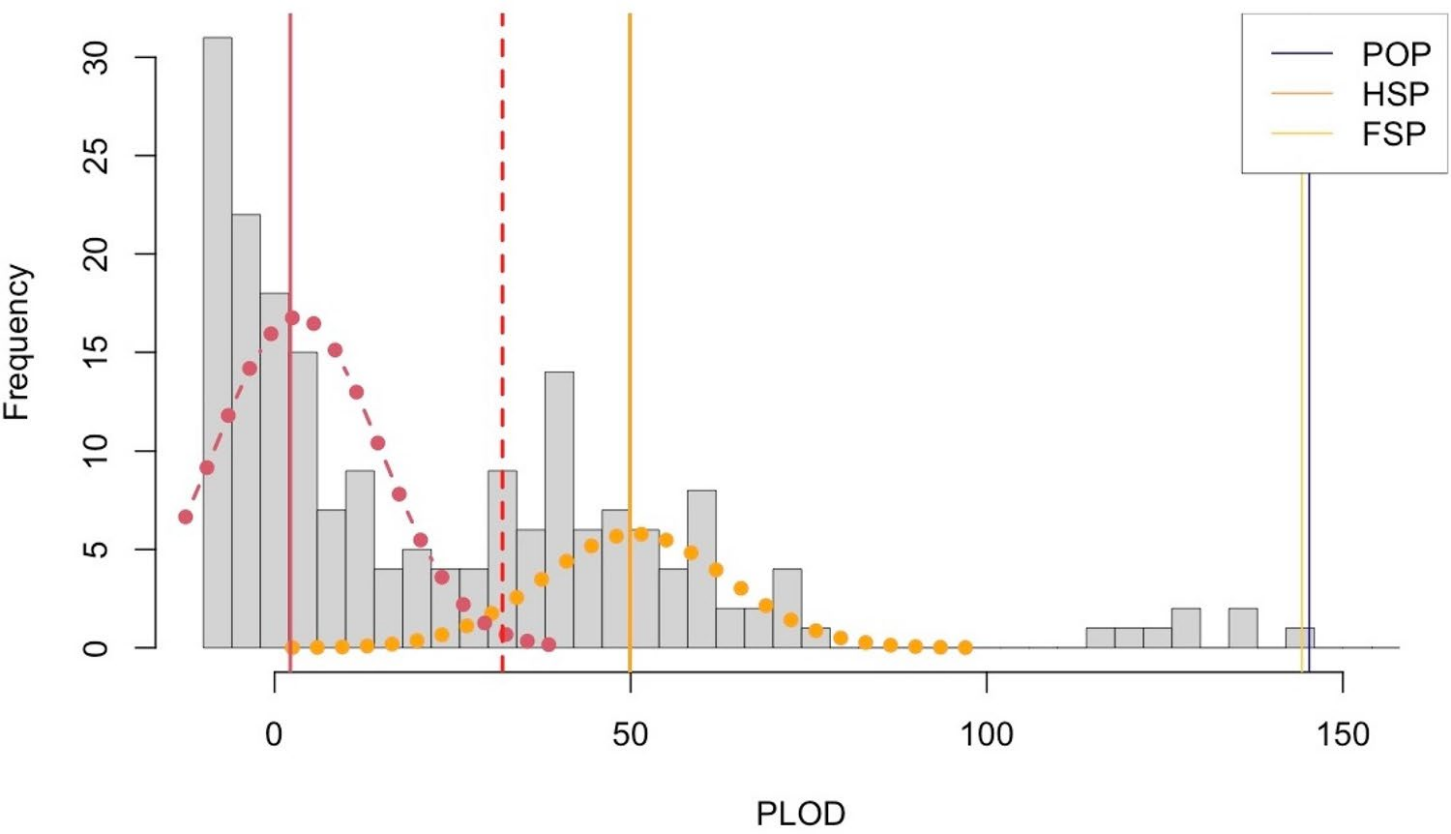
Frequencies of pseudo log-odds ratio (PLOD) scores at the expected half-sibling pair (HSP) and half aunt-uncle/niece-nephew pair region for comparisons between individuals less than 36 months old and cohort gap less than or equal to 5. The figure is a zoomed-in version of the total PLOD distribution (see Supplementary Fig. S1). The orange dotted line represents the normal approximation of the distribution of HSP PLODs and the orange vertical line represents the expected HSP PLOD peak, which aligns with the observed data peak. The red solid vertical line shows the expected PLOD for HTPs. The red dotted line represents the normal approximation of the distribution of HTP PLODs. The red dashed line is at PLOD value 32, which is the threshold used to ensure that less than 1 HTP lies above this value. The cluster at the right tail of the distribution contains the parent–offspring pairs (POPs) and full-sibling pairs (FSPs) with the vertical lines at PLOD score at approximately 150 showing the expected values for POPs (blue) and FSPs (yellow).

Summaries of the PLOD distribution for all pairwise comparisons between the 409 individuals present after quality control showed visible histogram peaks corresponding to theoretical expectations for unrelated, second-degree and first-degree relatives (Supplementary Fig. S1). A peak in the far-right tail of the PLOD statistic distribution indicates the presence of first-degree relatives, i.e., potentially full-sibling pairs (FSPs) and POPs, for a PLOD score greater than 100 (Fig. 1 and Supplementary Fig. S1). Fifteen pairs were detected with a PLOD statistic greater than 100 (Supplementary Table S2 summarises these pairs along with their associated covariate information. Note that Supplementary Table S2 also contains summaries for siblings described below). Discrimination of POPs and FSPs was investigated using a PLOD statistic specific for differentiating these kinship types (see “Methods”). Clear separation was observed between POPs and FSPs (Supplementary Fig. S2). The genetic discrimination of first-degree relatives was corroborated using age, individual sampling date, and mtDNA haplotype sharing information. Of the fifteen first-degree relative pairs, five showed strong evidence to be FSPs, two were mother–offspring pairs (MOPs) and eight father-offspring pairs (FOPs) (Supplementary Table S2). Under random mating we expect a priori FSPs to be rare as it requires the same mates to breed in subsequent mating seasons. If FSPs are not rare, then it implies a level of mate persistence, or potentially, fidelity to mating roost, or both.

Adult ages are unreliable for CIFFs older than 36 months (see ‘CIFF sample characteristics’ in “Methods”) and thus placing an older individual as the parent of a pair can be ambiguous. To avoid biasing the results by ‘informative censoring’, i.e., excluding data points a posteriori based on their outcomes rather than on their covariates, we excluded from the log-likelihood all POP comparisons (whether the outcome of the comparison was POP or not) that met the following criteria. Adult-adult comparisons where the age of the potential offspring was greater than 36 months because adult ages were unreliable above 36 months and the placement of the offspring relative to the parent ambiguous. We further excluded comparisons between individuals where the older individual of the pair (earlier birth year) was caught as a juvenile and could not have been old enough to be an adult at the birth of the juvenile. Given we made the modelling assumption that age at sexual maturity is 24 months (based on female value reported in Todd et al.^40^), we further excluded POP comparisons where the cohort gap was less than 24 months. Four POPs had a birth cohort age difference of 24 months or less (Supplementary Table S2). Possible explanations for this observation were age classification uncertainty for individuals older than 36 months and variation in the age at sexual maturity across individuals. Age at maturity and sexual maturity are different for CIFF and furthermore are different between the sexes (age at maturity is 27 months for males^40^).

For FSPs, two pairs were removed as recorded age was greater than 36 months for at least one individual in the pair. To include FSPs in the CKMR analysis the birth year of both individuals in the pair must be accurate, which is why these exclusions were made. We note that for modelling, comparisons for potential siblings were only made between individuals that have age measurements of 36 months or less.

Overall, there were six pairs that were very likely POPs as their birth cohort gap is greater than or equal to three years. Of these six, five had a male as the parent and one had a female. Two parent individuals, 6- and 4-year-old males (ID—65725, ID—67296), appeared twice implying that the offspring are siblings. These potential siblings were validated as a HSP and FSP. Therefore, for first degree relatives, we had one mother–offspring pair (MOP), five father-offspring pairs (FOPs) and three FSPs available for analysis.

### Half-sibling pairs

The set of second-degree relative pairs and potential half-sibling pairs were distributed around a centre-right peak of the PLOD distribution (Fig. 1 and Supplementary Fig. S1). The distribution of second-degree relatives contained two sources of unwanted relative pairs that have different methods for exclusion: (1) pairs of second-order relatives with the same expected PLOD score as HSPs including grandparent-grandchild (GGP), and aunt-uncle/niece-nephew pairs; (2) contamination of the lower tail of the distribution with less related pairs e.g., cousins and half aunt-uncle/niece-nephew pairs. A detailed description of the procedure used to remove these unwanted kin pairs is presented in Supplementary Note B.

Following these procedures 65 HSPs were detected. Aunt/uncle-niece/nephew pairs can be arbitrarily close in cohort gap but require FSPs to frequently persist in the population into adulthood for this type of relationship to contaminate the HSP distribution. We performed a simulation study to investigate the rate of aunt/uncle-niece/nephew pairs across different scenarios of mate persistence and found only a small proportion, close to the number of FSPs, in scenarios similar to CIFF observations (see Supplementary Note C). If present for CIFF, the retention of these aunt-uncle/niece-nephew pairs as HSPs would lead to a downward bias of reported abundance estimates approximately proportional to the fraction of HSPs, therefore, approximately 3/65 $\approx$ 0.045.

The age gaps between the pair of individuals in each HSP ranged between zero and six years with 20 having the same birth cohort (Table 1). This observation could not be driven by females as they can only have at most one pup per year and thus any one female cannot be the shared parent of any two juveniles born in the same birthing season. Assuming age classification is accurate, if two juveniles born in the same year are a HSP then they must share a father. This large number of same birth cohort HSPs coupled with the information that two POPs are from the same father and the presence of FSPs, strongly suggested male reproductive skew and mate persistence for CIFF.

**Table 1:** Summary of first- and second-degree kin detected.

Year gap	0	1	2	3	4	5	6	Total
Parent-offspring								
Maternal	–	0/3644	0/3965	1/2992	0/2312	0/429	0/460	1/13,802
Paternal	–	3/10,119	2/7962	0/5153	0/3726	0/650	0/713	5/28,323
Siblings								
Full	–/–	1/20,718	2/14,660	0/6864	0/2250	0/759	–	3/58,311
Half	20/13,060	21/20,718	16/14,660	5/6864	2/2250	1/759	–	65/58,311

Year gap corresponds to the difference between the birth year of the juvenile and the capture year of the adult for parent–offspring pairs and the two birth cohort years for sibling pairs. Each cell shows the number of kin and the number of pairwise comparisons made to detect these kin (kin/pairwise comparisons). The siblings row summarises the full-sibling pairs (three) and half-sibling pairs (65) detected in the analysis. The cells with a '–' entry are differentiated from a 0 entry to indicate that this observation was not considered for CIFF CKMR analysis.

The ratio of males to females (Supplementary Table S1) in the total sample in the juvenile age class, which should be less prone to selective sampling, was approximately 1.6:1 male to female individuals. This implied that it is unlikely that these HSP/FSP observations are due to a male population that is smaller than the female population and with some males having to breed multiple times for all females to have a partner.

### Mitochondrial DNA sequencing

Given the different reproductive dynamics of the sexes, a sex specific CKMR model was required. To infer the sex of the shared parent for the HSP component of the model, we used mitochondrial DNA (mtDNA) sequencing. This was performed for the set of individuals in the pairs that showed evidence for first or second-degree relative status (see Supplementary Note D for details). It was expected that the CIFF has enough mtDNA diversity in the mtDNA control region sequenced based on prior studies^39^.

Across the quality-controlled mtDNA sequences, 65 unique haplotypes were observed. The mode of the haplotype frequencies was 1.1% with one haplotype showing a frequency of ≈ 20% (Supplementary Table S2 and Fig. S3). For each pair with a PLOD score greater than 32, we determined if individuals in the pair shared the same haplotype and the frequency of the haplotype assigned to each individual in the pair (Supplementary Table S2). Individuals involved in FOPs did not share mtDNA haplotypes, except one pair that was excluded prior on ageing criteria. Depending on the frequency of haplotypes it is possible that FOPs share a mtDNA haplotype by chance. Across the first-degree relative set, the single MOP and viable FSPs showed identical mtDNA haplotypes.

For the 65 HSPs, 17 of the pairs shared mtDNA haplotypes, 28 of the pairs had different (or could not be differentiated) mtDNA haplotypes and a birth cohort gap of at least one year, and 20 pairs had different mtDNA haplotypes and the same birth cohort (intra cohort). Three of the HSPs (one pair intra and two inter) could not be differentiated based on mtDNA as one of the individuals of each pair did not have an mtDNA sequence available due to laboratory quality control failure.

### CIFF CKMR modelling

The genetic kinship analyses strongly indicated higher reproductive success for a subset of males across the breeding seasons. Furthermore, the presence of FSPs suggested there was the potential for mate persistence. These initial observations implied that learning quantitatively from CKMR about CIFF male demography was more challenging because the estimates of male abundance and survival were potentially confounded with the rate of dominant male turnover.

Given female CIFF have at most one pup per year, we initially grounded our results using a female-focussed CKMR model because it was simpler than the model including both sexes, and we were more confident with the female-related biological assumptions (see “Methods” and Supplementary Note G for a detailed description of the female-focussed model components). Briefly, the female-focussed model describes the number of female adults ${N}_{{\female},t}{ p}$resent at an arbitrary unit of discrete-time *t*, taken to be years from here on, by the function

$${N}_{{\female}, t}={N}_{{\female},{t}_{0}}\mathrm{exp}\left[{\rho }_{{\female}}\left(t-{t}_{0}\right)\right],$$

where ${N}_{{\female},{t}_{0}}$ is the adult female abundance at the initial year of modelling *t*_0_, and ${\rho }_{{\female}}$ is the female population rate of increase. The kinship probabilities for both POPs and HSPs depend on adult female abundance at time *t* and the survival function that specifies the survival rate of an individual between time ${t}_{1}$ and ${t}_{2}$ (${t}_{1}$< ${t}_{2}$) and is governed by the mortality rate *δ* via $\varphi ({t}_{1},{t}_{2}) =\mathrm{ exp}[-\delta ({t}_{2}- {t}_{1})] (\mathrm{see\;Methods})$. Therefore, for CIFF, the female-focussed CKMR demographic model contains three parameters ${\varvec{\theta}}$ $={\left({N}_{{\female},{t}_{0}}, {\rho }_{{\female}}, {\delta }_{{\female}}\right)}{\prime}$. Initial investigations indicated that mortality may be estimated with poor accuracy given the available data. We, therefore, fitted the female-focussed model with the mortality parameter constrained through a prior distribution that was informed from estimates of survival from a previous MR study^42^ (see “Methods” and Supplementary Note H for details on the prior distribution).

The CKMR model was then extended to include male population dynamics, male reproductive skew and mate persistence. The male population estimates were of high ecological interest and the model is more complete relative to the female-focussed model, which required some simplifying assumptions to fit without the male dynamics (see Supplementary Note G). In outline, the population dynamics for total adult abundance was modelled as

$${N}_{t}={N}_{{\female},{t}_{0}}\mathrm{exp}\left[{\rho }_{{\female},{\male}}\left(t-{t}_{0}\right)\right] + {N}_{{\male},{t}_{0}}\mathrm{exp}\left[{\rho }_{{\female},{\male}}\left(t-{t}_{0}\right)\right],$$

$\mathrm{where\;} {N}_{{\female},{t}_{0}}$ and ${N}_{{\male},{t}_{0}}$ are the adult female and adult male abundance at the initial year of modelling *t*_0_, and ${\rho }_{{\female},{\male}}$ is now a common trend parameter for adult females and males. In the extended models a sibling probability was computed by summing over three demographic probabilities FSP, MHSP, and PHSP, where the FSP probability incorporates a parameter *α* that models mate persistence (see “Methods” for details). To incorporate male reproductive skew, we make the distinction between the total set of adult males and the breeding set of males by incorporating the breeding male population size in the computation via ${N}_{{\male},{t}_{0}}\times \pi$, where $\pi$ lies in (0*,* 1). The parameter set estimated for the base extended model was ${\varvec{\theta}}$ $={\left({N}_{{\female},{t}_{0}}, { N}_{{\male},{t}_{0}}, {\rho }_{{\female},{\male}}, { \delta }_{{\female},{\male}, } \alpha , \pi \right)}{^\prime}$ (referred to as the 'base extended' model).

We explored three further variants of the base model. The second extended model incorporates a prior on the shared mortality rate (${\delta }_{{\female},{\male}, })$ as detailed above for the female-focussed model (referred to as the 'base extended + prior' model). The third extended model include an extra mortality parameter ${(\delta }_{{\male}* })$ that is included in the paternal sibling calculations ('base extended + mortality'). The final includes the extra mortality parameter and a prior on the shared mortality parameter ('base extended + prior + mortality').

All available kin and mtDNA information were included in the proposed CKMR models and parameter estimates obtained via maximising the penalised log pseudo-likelihood (implemented as a covariate-class Binomial log pseudo-likelihood (see Supplementary Eq. S12).

In the main text, we present the estimates from the base extended model. The results of the female-focussed model and all four extended models were summarised in Supplementary Notes G and H. For each of the extended models, the elements of the gradient vector at the MLE estimates was close to zero indicating adequate convergence of the optimisation algorithm. Fit was similar across models based on the observed versus expected kin i.e., all observed kin were within the 95% interval of the Poisson distribution generated by the expected number of kin for each of the cells in Table 2 and Supplementary Table S7 (see “Methods” for explanation of Poisson approximation). Goodness of fit measures were similar across extended models with the base extended model showing marginally the lowest value (Supplementary Table S7).

**Table 2:** Summary of observed versus expected kin.

Year gap	0	1	2	3	4	5	6	Total
Parent–offspring maternal	–	0/1.47	0/1.36	1/0.860	0/0.560	0/0.080	0/0.070	1/4.41
Paternal	–	3/2.16	2/1.45	0/0.780	0/0.480	0/0.070	0/0.060	5/5.00
Siblings full	–	1/1.55	2/0.77	0/0.26	0/0.060	0/0.010	–	3/2.64
Maternal half	–	6/6.53	6/4.00	3/1.61	2/0.440	0/0.130	–	17/12.7
Paternal half	20/–	15/17.2	10/10.3	2/4.08	0/1.11	1/0.320	–	28/33.1

Year gap corresponds to the difference between the birth year of the juvenile and the capture year of the adult for parent–offspring pairs and the two birth cohort years for sibling pairs. Each cell shows the number of observed kin versus (/) the expected number of kin, which is computed as the number of combinations multiplied by the kinship probability computed under the maximum likelihood estimates of ${\varvec{\theta}}$. The siblings' row is differentiated by sex for half-sibling pairs by taking those individuals that share a mitochondrial DNA haplotype as maternal for illustration.

**Figure 2:**
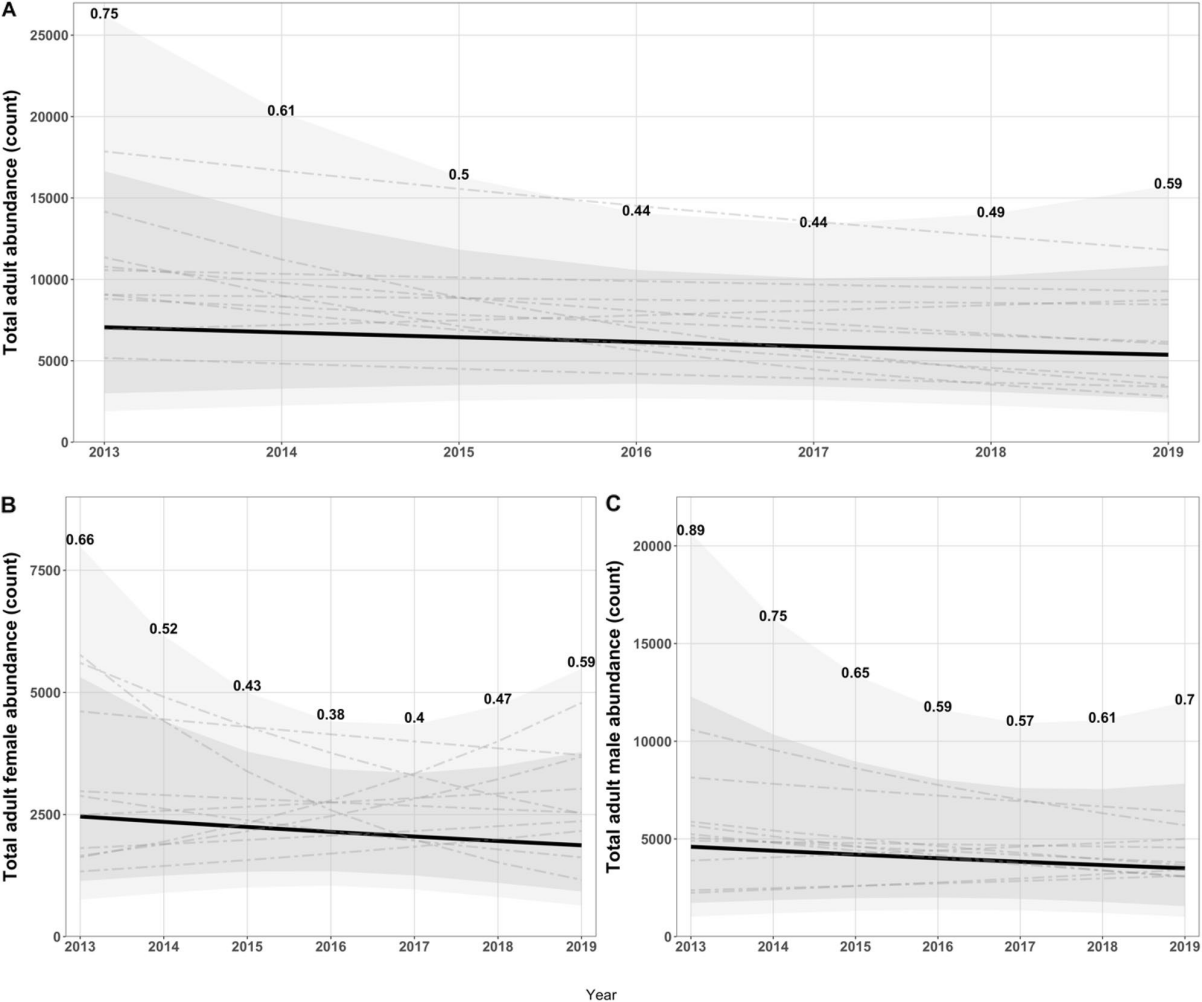
Estimated trend in adult abundance. Estimated time series (dark solid line) of total adult (**A**), adult female (**B**) and adult male (**C**) abundance estimated from the close-kin mark-recapture base extended model. Confidence intervals are depicted with grey shaded areas with 95% external and 80% internal. The coefficient of variation for each year’s abundance estimate is reported as bold values above each upper interval estimate. Dotted lines are ten sampling importance resampled lines added to show variability in trends generated from parameter estimates and their variability.

The adult female abundance estimates in the base extended model were reasonably precise with the highest precision estimates being in years 2016 and 2017 (Supplementary Table S8 and Supplementary Figs. S10 and S11). For 2017, adult female abundance was estimated as 2049, with a 95% confidence interval (CI) of 967–4340 and coefficient of variation (CV) of approximately 40% (Table 3 and Fig. 2). Profile likelihood 95% confidence intervals for adult abundance were similar to those estimated using the Hessian standard errors (Supplementary Table S8 and Supplementary Fig. S12). Similar results from the female-focussed model were observed with 1948 adult females estimated in 2017 and a similar CV (Supplementary Table S5). Although similar, estimates of trend and mortality are difficult to compare between the female-focussed and extended models because they are tracking both sexes in the extended models. In the base extended model, total adult abundance had a point estimate of 5883 (SE = 2468) in 2016 with an 95% CI interval of 2585–13,385. Across models the lower bound on total adult abundance was relatively consistent at $\mathrm{approximately}$ 2200–2500 individuals (Supplementary Table S8).

**Table 3:** Summary of base extended CKMR model parameter estimates and uncertainty.

Parameter	Estimate	SE	95% CI	
			Lower	Upper
${N}_{{\female},{t}_{0}}$	2459	1478	753	7987
${N}_{{\male},{t}_{0}}$	4601	3527	1024	20,669
${N}_{{\female},{t}_{2017}}$	2049	785	967	4340
${N}_{{\male},{t}_{2017}}$	3834	2048	1346	10,921
${N}_{{t}_{2017}}$	5883	2468	2585	13,385
${\rho }_{{\female},{\male}}$	− 0.046	0.148	− 0.335	0.244
${\delta }_{{\female},{\male}}$	0.198	0.159	0.041	0.952
$\alpha$	5.435	0.563	4.332	6.538
$\pi$	0.230	0.115	0.077	0.515

Hessian-based standard errors (SE) are reported from TMB with 95% confidence intervals using *z*_1−0*.*05*/*2_ as the SE multiplier constructed and then transformed for those parameters estimated on a transformed scale. The parameters ${N}_{{\female},{t}_{0}}$, ${N}_{{\male},{t}_{0}}$ are the abundance estimate in the first year of modelling (2013) for females and males respectively. Estimates of ${N}_{{\female},{t}_{2017}}, {N}_{{\male},{t}_{2017}}$ and ${N}_{{t}_{2017}}$ correspond to the female, male and total adult abundance in the lowest coefficient of variation year. Parameters ${\rho }_{{\female},{\male}}$ and ${\delta }_{{\female},{\male}}$ correspond to the adult trend and mortality parameters estimated together for females and males. Quantities $\alpha$ and $\pi$ model mate persistence and the proportion of the total adult male abundance contributing to reproduction respectively.

Graphs of kin with the same mt-DNA haplotype (including MOPs which are not ambiguous based on mt-DNA) showed very few clusters of kin, which implies that when performing any one comparison the knowledge of all prior same-haplotype comparisons provides little information on whether the comparison will be a same-haplotype kin pair (Supplementary Fig. S13). This implies that the assumption of pairwise independence of comparisons is reasonable for female parameters and variance estimates should be reliable. Clusters were more apparent for kin with different mt-DNA haplotypes (Supplementary Fig. S14). Therefore, the uncertainty estimates for male-associated parameters may be underestimated based on the Hessian and profile likelihood methods and should be interpreted with more caution.

The estimated time series of total adult, adult female, and adult male abundance shows an expected depreciation in precision at the edges of the time horizon modelled, with the largest variability observed in 2013 (first year modelled) (Fig. 2). The point estimate for the trend in total adult abundance was mildly decreasing but highly variable, with the data being consistent with a trend in adult abundance potentially increasing or decreasing (Table 3 and Fig. 2). The sampling importance resampling (SIR) trends (see “Methods”) showed this well, with many of the 10 sampled trends increasing (Fig. 2). The estimate of the average annual adult mortality rate was 0.198 (SE = 0.159), which was not substantially different from the value estimated in the base extended + prior model (0.291 (SE = 0.129)), which suggests that sex-averaged survival may be higher than previous estimates (Supplementary Table S8). Precision for the mortality rate estimates was poor and should be interpreted cautiously. Hessian and profile likelihood standard errors were discrepant for the mortality rate parameter with substantial asymmetry on the lower bound across the extended models (Supplementary Table S8 and Supplementary Fig. S12). The use of a prior on mortality rate assisted with constraining uncertainty in the lower bound, which was observed in results from the profile likelihood interval method (Supplementary Table S8). For the base extended + extra mortality model, the point estimate for ${\delta }_{{\female},{\male}}$ decreased mildly to 0.149 and the estimate for the breeding males was 0.244 (SE = 0.218). For the base extended + prior + mortality model, a similar point estimate for ${\delta }_{{\male}*}$ was observed when a prior was used for ${\delta }_{{\female},{\male}}$. For both base extended + mortality and base extended + prior + mortality models, uncertainty in the mortality parameters was high and there was little evidence for difference in these parameters. The inclusion of these extra parameters led to a marginally poorer fit based on the goodness of fit measures when compared to the base model (Supplementary Table S7).

The estimate of the breeding male proportion (of total adult male abundance) was $\approx$ 20% across all models with an estimate of 0.230 (SE = 0.115) in the base extended model. The point estimates for *α* were consistently $\approx$ 5.4 (SE = 0.563 in base extended) across models, which suggests that there is strong evidence of mate persistence, leading to an inflated rate of full sibling pairs in the CIFF population. We took ratios of the FSP probability (see Eq. (6) of “Methods”) across covariate classes computed at the reported estimates for the base extended model to the probability under a scenario of a random selection of mates (i.e., *α* = 0). An *α* = 5.435 corresponded to an approximate 200 times increase in the FSP probability on average across covariate classes.

## Discussion

The critically endangered Christmas Island flying-fox is a priority for conservation management, and its apparent decline raises serious concerns about both its own survival and the keystone ecological services it provides as the sole remaining native mammal on Christmas Island. To direct the CIFF effectively away from extinction, management requires accurate estimates of population demographics and dynamics. To this end, we extended the CKMR method to estimate sex-specific abundance, survival, and trend for this species. This work is the first application of CKMR to a terrestrial species and shows its capacity as a tool for monitoring threatened species.

The genetic kin-finding analysis of 409 individuals showed that first and second-order kin could be reliably detected for CIFF based on SNP markers genotyped using DArTseq. Overall, six parent-offspring, three full-sibling and 65 half-sibling pairs were valid for CKMR modelling. The CKMR method revealed vital information on survival, trend, and the CIFF mating system, including strong evidence of skewed male reproductive success. This skew makes quantification of the adult male census population via CKMR challenging because abundance is linked to the breeding set of males and survival is confounded with the rate at which dominant males exit the breeding population. We overcame these challenges through extending the CKMR theory and estimated total adult abundance to be ≈ 5900 (95% CI 2600–13,500) individuals for the highest-precision year. Estimates of total male abundance were ≈ 3,800 (95% CI 1350–10,900). However, the average proportion of this estimate that were breeding was 23% suggesting a point estimate of ≈ 750 males breeding in 2017 with a 95% CI of 450–1730. Kin clusters were more apparent for kin with different mt-DNA haplotypes, which implies that the pairwise independence assumption of the pseudo-likelihood is unreliable for male-derived parameters. This does not bias the point estimates but does affect the variance estimates of the male parameters^10,43^. Variances may be underestimated based on the Hessian and profile likelihood methods and should be interpreted with more caution. Quantifying the extent of the underestimation is an interesting and challenging topic, which we leave as future work. This is anticipated to arise frequently in studying endangered wild populations. We expect variances of female parameters to be well estimated. Adult female abundance was estimated to be ≈ 2,050 individuals in the high precision 2017 year with a 95% CI of 960–4350. Adult female abundance speaks directly to the size of the effective breeding population and hence capacity for population growth, and so is arguably the most important component of the population to estimate.

Our estimates indicate that the present CIFF population is larger than the 1000–2000 individuals estimated in the early 2000s^15,44^. CIFF nocturnal distance sampling surveys conducted between 2006 and 2012 reported a population estimate of 2100 (S.E. 563)^17^ and a decrease in CIFF incidence over the occupancy survey period^16^ relative to the population estimate of 6000 from Tidemann^20^. However, results from a recent MR study indicated an increasing trend in the estimated population size from 2430 (95% CI 1665–3868) in 2016 to 3846 (95% CI 3451–4309) individuals in 2018^42^. The point estimate of total adult abundance from CKMR was larger, but CKMR intervals included the MR point estimates. The CKMR estimates may be downwardly biased by contamination of the HSP set with aunt-uncle/niece-nephew kin. We expect the bias, if present, to be approximately proportional to the fraction of HSPs. The simulation indicated that the number of aunt-uncle/niece-nephew relationships to be close to the number of FSPs so approximately 3/65 $\approx$ 0.045, which is well within one standard deviation of the presented uncertainty of the presented estimates. CKMR abundance estimates integrate uncertainty over an expanded parameter set and lost 40% of the samples through laboratory quality control, making comparisons of uncertainty per unit effort between these studies difficult. The CKMR point estimate of trend in adult female abundance indicated a mildly decreasing population, but the precision of the estimate was poor and could neither exclude nor corroborate the trend results of Todd^42^. Caution should be exercised when interpreting the differences between recent population size estimates and those of the early 2000s because the underlying model assumptions and data used are difficult to reconcile between these studies. In either case, an estimate of adult abundance that is larger than previously reported is a positive sign for the population health of the species.

The CKMR method revealed additional information on the CIFF mating system, which was driven by the primary observation that 20 sibling pairs were intracohort. McCracken and Wilkinson^45^ remarked that there is no convincing evidence that mating in any bat species is random and that bats exhibit mating systems ranging from monogamy to classic lek mating systems. Across members of the *Pteropus* genus there is evidence of polygyny both in the wild and in captivity^45^. *Pteropus rodricensis* forms apparent harem groups of one male with up to eight adult females^46^. *Pteropus hypomelanus*, *P. vampyrus* and *P. pumilus* appear to mate in small harems in captivity^45^. Todd et al.^40^ concluded that CIFF has a polygynous mating system with breeding being seasonal and annual. Tidemann^20^ also observed that a small proportion of the male population monopolises all adult females. Personal observations of harem structures were recorded at CIFF roost sites during recent sample collection, which further suggests a skew in mating opportunities. As female CIFF can have at most one pup per year, the 20 observed intracohort HSPs are likely from males that have mated with multiple females in a breeding season. Mitochondrial DNA sequencing indicated that the shared parent for these intra-cohort siblings was very likely male. This is also evidence for reliable age classification as female intra-cohort pairs would be present if ageing errors occurred although intra-cohort pairs can, with a small probability, share a mtDNA haplotype and still share a father. We further observed that two of the five paternal POPs were from the same father. Individual male reproductive success may vary around an average of one mate from year to year, which could produce substantial numbers of intra-cohort pairs without any one set of individuals dominating year-on-year. Quantitatively the base extended CKMR model estimated that ≈ 23% (95% CI of 0.077–0.515) of the total adult male population contributed to breeding on average over the years studied. The estimated reproductive skew provides useful knowledge to inform conservation management actions, including population viability models.

We further observed three FSPs, which under random mating we expect to be very rare as it requires the same individuals to meet in subsequent mating seasons. The mate persistence parameter (*α*) was estimated to be substantially non-zero indicating an inflated rate of mate persistence relative to individuals randomly choosing new partners in each new breeding season. While interseason mate fidelity has been documented in other bats^47^, to date it has not been observed in *Pteropus* species in the wild. There is the potential for site fidelity to generate FSPs with females potentially mating with the same mate by association with the mating territory; however, few studies have examined this in *Pteropus* species and Welbergen^48^ showed that *P. poliocephalus* females join a male's mating territory but showed limited mate persistence within years and none between years. Combined, both prior and CKMR observations and estimates point strongly to, at least in part, a single-male, multi-female group mating system for CIFF with the potential for mate persistence, roost fidelity, or both.

Mitochondrial DNA was critical to the implementation of the female-focussed and extended CKMR models as it allowed the substantial set of sibling pairs detected to be partitioned by the sex of the shared parent. Information on mortality and trend in CKMR needs several juvenile cohorts to estimate these parameters, which are based on how fast the proportion of siblings declines as the number of cohorts separating them increases. The approximate point estimate for the averaged adult female and male survival was 0.82, which is larger than the adult female and male survival reported in Todd^42^ of 0.65. When the breeding male mortality was modelled separately the point estimate for the common mortality parameter decreased. The point estimate of breeding male mortality was greater suggesting lower survival, which may reflect a greater stress on breeding males. We note that across models the precision was too poor to make reliable inference about differences in point estimates of survival. However, if sampling of CIFF for CKMR were to continue for the CIFF we expect the precision of mortality/survival estimates to increase rapidly as the numbers of kin and differentiated cohorts increases.

The kinship analysis showed peaks in the PLOD kinship statistic that aligned well with theoretical expectations and variability. However, the PLOD distribution was not as well spread as for other species, e.g., Atlantic bluefin tuna^43^. For CKMR analyses of other species, studies have used a variant of DArTSeq namely DArTCap, which gives much higher sequencing read depth per locus than DArTSeq, and lower genotyping error rates, which can improve the spread of the PLOD distribution and in turn discrimination of kinship classes. DArTCap requires knowledge of the variants intended to be sequenced and has an initialisation step to manufacture the capture probes that bind to the restriction fragments for sequencing^49^. The SNP markers used in this study could be used for this initialisation step and once performed DArTCap offers a cheaper and more reliable genotyping platform than DArTSeq for future monitoring of CIFF.

An important research goal for CIFF and other threatened species would be to develop accurate ageing methods. This would facilitate adult-adult comparisons in the analysis and an increase in kin detected per sample. Although ageing by tooth extraction and examination has been conducted in surveys of other flying-fox species^50^, the method can have implications for animal welfare and fitness. Thus, a more suitable method for age determination could include epigenetic age estimates^51^ from the biopsy used for CKMR, or the use of the universal mammalian epigenetic age predictor^52^. The presence of a high-quality assembled CIFF genome is under consideration and could, in principle, make CKMR more efficient through the detection of more distant kin and assist the development of cheaper genotyping. A powerful feature of the CKMR method is that subsequent sampling improves the precision of the current analysis as new samples can be compared to all previous samples, offering a cost-effective and accurate continuous monitoring method. The limits to precision by continuous monitoring, which are influenced by generation length and sampling intensity (among other elements of the species' biology), can be explored through CKMR design principles. Further analyses of minimum sampling requirements for CKMR monitoring maintenance could be performed given the results from this larger study using the theory outlined in Bravington et al.^10^. Crucially, whatever the particulars of future CIFF sampling, CKMR for CIFF only requires DNA samples and avoids the maintenance and ethical issues associated with a marked population. CKMR methods can be integrated with existing MR monitoring, providing additional detailed understanding at a low cost. The findings of CKMR analyses offer important insights particularly for threatened species.

This study is the first application of CKMR to a threatened terrestrial mammal. The study provided state-of-the-art estimates of key female and male demographic parameters, revealed further insights into the CIFF mating system, and, through a novel model, estimated the extent of male reproductive skew and mate persistence in this sample. This study provides pivotal abundance and trend estimates that together with the recent MR study form a sound baseline for ongoing management of CIFF.

CKMR has clear potential for understanding the demography of mammals and as a broad tool for the monitoring and management of threatened terrestrial species. Future applications of CKMR to terrestrial species will encounter similar challenges to those overcome in this study, for example, polygamy, reproductive skew, mate persistence and sex-specific demography. A substantial challenge, not encountered in this study, that will arise for many species is limited dispersal. Spatially explicit CKMR models have been explored^30,53^ to address this issue with the added model complexity needing consideration for many species. We anticipate that the techniques detailed in this work will serve as a methodological reference point for many future applications and extensions to terrestrial species, which is critical to the uptake and dissemination of the powerful CKMR method.

## Methods

### Sampling and tissue collection

Christmas Island is a territory of Australia located in the Indian Ocean approximately 380 km south of Java, Indonesia, and 1500 km west of the Australian mainland. It is a small 135 km^2^ island composed of tertiary limestone overlying volcanic andesite and basalt and its topography consists of a series of limestone terraces separated by cliffs^20^. Samples and measurements from individual CIFF were collected across the island during multiple sampling exercises for several different studies^19,24,39,40^. Those studies detail the capture, morphological measurements, and tissue sampling procedure of the samples included in this analysis. Briefly, CIFFs were caught using nets at both foraging and roost sites between August 2015 and February 2019. Capture locations were distributed across the island and the net location within each roost site was randomly selected. Foraging sites were chosen using night driving surveys from random starting locations and fruiting trees selected as a capture site were random on any given night^40^.

Captured animals were processed at the capture site where they were anaesthetised and sex and age determined, and morphological measurements recorded; one of the capture sites was a field laboratory (The ‘Pink House’ building in Christmas Island National Park). Age class was determined using morphologic measurements and secondary sex characteristics, including tooth staining and wear and in females, teat elongation. For each individual, wing-membrane biopsies were taken and stored in ethanol for genetic analyses.

#### Approval for animal experiments

The research protocols used across the fieldwork adhered to the guidelines of the American Society of Mammalogists for research on live mammals^54^ with approval from the Animal Care and Ethics Committee of Western Sydney University (Project Protocol No. A11140). Permits to capture, handle, band and measure bats were issued by the Christmas Island National Park (Permit No. CINP-2015-6-1).

### Kin-pair detection

To genetically detect kin pairs, we performed single nucleotide polymorphism (SNP) genotyping for each available CIFF individual using DArTseq. The tissue preparation, genotyping and quality control procedures used are detailed in Supplementary Note A.

To estimate kinship, the probability of the two observed genotypes at each locus for a pair of individuals is computed as if the pair was truly a HSP versus truly an unrelated pair. The likelihood ratio under these two kinship categories was computed from these two probabilities using functions in the Kinference R package for all loci and the mean of the log-ratios is taken as the overall statistic for the pair and is referred to as the pseudo-log-odds-ratio (PLOD). Large positive values are evidence for HSP status and negative values are evidence against; full-sibling pairs (FSPs) and parent–offspring pairs also have very high positive PLOD scores and can be separated from half-siblings because their expected PLOD can be calculated a priori. Bravington et al.^10^ and Hillary et al.^11^ detail further the computation of the likelihood ratio for different kinship types. PLOD statistics were computed for each pair of individuals and the distribution was summarised to infer kinship.

Statistical genetics theory was used to predict the expectation and variance of the distributions of different kinship types, including parent–offspring pairs (POPs), full-sibling pairs (FSPs) and half-sibling pairs (HSPs) (Supplementary Fig. S1). These theoretical predictions were compared with the observed distributions to assess the adequacy of the genotype set used for kin finding and then subsequently to determine pairs that are very likely to be of a particular relatedness type, e.g., POPs, FSPs and HSPs.

First degree relatives including FSPs and POPs also have very high positive PLOD scores and are typically separated from second-degree and lower order kin by simple segmentation of the PLOD x-axis. Discrimination of POPs and FSPs was investigated using a PLOD statistic specific for differentiating these kinship types implemented in the split_FSPS_from_POPs function the Kinference R package and is robust to genotyping error. Again, the probability of the two observed genotypes at each locus for a pair of individuals is computed as if the pair was truly a POP versus truly an FSP and the summed log likelihood ratio computed. Discrimination of FSPs from POPs is based on inspection of the distributions of the PLOD for clear separation of the two kinship types and comparison against their expected values.

The set of second-degree relatives is determined by inspecting the pairs whose PLOD distribution is centred at the HSP expectation. This distribution contains HSPs and unwanted relative pairs that have (1) the same expected PLOD score (e.g., grandparent-grandchild) and (2) contamination from less related pairs (e.g., cousins). A detailed description of the procedure used to segment second-degree relatives and remove contamination in the lower tail from lower-degree relatives is presented in Supplementary Note B.

### CIFF sample characteristics

Phenotypic information for each CIFF sample included an individual identifier, collection date, capture location, sex, forearm and tibia length, developmental stage i.e., juvenile, sub-adult and adult and an estimate of age. For CKMR analysis, age was divided into three broad classes including juvenile (0–12 months old), sub-adult (> 12–24 months old), and adults (> 24 months old). Age determination for CIFFs older than 36 months is more difficult; once an individual becomes an adult, age can be estimated but a discrepancy between the age estimate and actual age of ± one year is likely especially for individuals past 36 months. Therefore, age is reported for each individual but only juveniles, sub-adults and adults less than 36 months (third year of life) old were considered to have a known age. Age at sexual maturity is assumed to be 24 months, which corresponds to the age of female sexual maturity reported in Todd et al.^40^. Across the set of 409 individuals that passed genotyping and kinship analysis quality control, 176 were less than 12 months old, 104 were $\ge$ 12–24 months old, 62 $\ge$ 24–36 months old, and 66 were $\ge$ 36 months old. In total, the years of sampling ranged from 2015 to 2019 with 46, 158, 58, 48, and 99 individuals sampled in each year respectively.

### CIFF biology

The biology of the CIFF was used to inform the computation of the CKMR kinship probabilities. The findings of Todd et al.^40^ indicate that most births occur between the months of January and April with the peak frequency of births occurring in February and March and conception occurring between June and August. Todd et al.^40^ noted that mating and birth were not limited to these peak periods and occurred with substantial frequency throughout much of the year. Therefore, we grouped individuals by their likely natural birth cohort rather than calendar year cohort—for example, if an individual was estimated to be one year old and caught in December 2017 then we considered it to be from the 2017 cohort rather than the calendar year cohort of 2016.

The small size of Christmas Island (135 km^2^ total area and 22 km maximum width) and the flying speed of the CIFF imply that individuals can potentially disperse to any location within a night. The very large distances to any other potential habitats outside of Christmas Island further imply that the CIFF population is spatially circumscribed with minimal emigration. We assume that for CIFF, fecundity is constant with females having one pup per year, the age of maturity is 24 months, and there is no stock structure. Kin detection can assist us in checking the validity of these assumptions, but we assume this to be the base model scenario.

The CIFF shows evidence for a polygamous mating system with breeding being seasonal and annual^40^. Tidemann^20^ suggested that a small proportion of the male population monopolises all adult females, with James et al.^15^ concluding that this hypothesis required further investigation. Initial genetic kinship analyses strongly indicated higher reproductive success for a subset of males across the breeding seasons. Furthermore, there was the potential for mate persistence indicated by the presence of FSPs. Given these initial observations, learning quantitatively from CKMR about CIFF male demography is more challenging because the estimates of male abundance and survival are confounded with the rate of dominant male turnover. However, given female CIFF show evidence for having at most one pup per year, models focusing on females are anticipated to provide reliable information about female CIFF demography.

### CKMR method overview

For CKMR the focus is on pairwise comparisons of sampled individuals. Let $i$ and $j$ represent two individuals sampled from a population and the comparison of their genotypes generates a discrete kinship category ${K}_{ij}$ which, for example, could be unrelated, mother–offspring or half-sibling pair.

The probability of a particular kinship relationship, written as ${\mathbb{P}}({K}_{ij} = {k}_{ij }| {{\varvec{z}}}_{i},{{\varvec{z}}}_{j};{\varvec{\theta}}$) can be calculated for each pair and kinship type. The notation emphasizes that the probability is conditional on covariate vectors ${{\varvec{z}}}_{i}$ and ${{\varvec{z}}}_{j}$ gathered for the two individuals at capture, noting that the covariates ${{\varvec{z}}}_{i}$ and ${{\varvec{z}}}_{j}$ do not include information on genotype or kinship to other individuals. Common covariates include year of sampling, sex, and year of birth $({y}_{i},{y}_{j})$ if ages can be obtained. The kinship probabilities are the building blocks of the pseudo-likelihood defined below and link the population dynamics model, and the associated parameters ${\varvec{\theta}}$, with observed kinship for the pair of individuals. Typical base model parameters include adult abundance at time $t$, trend, and mortality/survival. For brevity in the following, we drop the ${\varvec{\theta}}$ from the notation.

To formulate expressions for the kinship probabilities, we use the notion of expected relative reproductive output (ERRO)^10^. For example, the probability that a sampled female $i$ is the mother of $j$ can be written as

$${P}_{ijk}={\mathbb{P}}({K}_{ij}= k | {{\varvec{z}}}_{i},{{\varvec{z}}}_{j})=\frac{{\mathbb{E}}\left[{R}_{i}\left({y}_{j}\right)|{{\varvec{z}}}_{i}\boldsymbol{ }, {{\varvec{z}}}_{j}\right]}{{\mathbb{E}}\left[{R}_{+}\left({y}_{j}\right)|{{\varvec{z}}}_{j}\right]},$$

where $k$ indexes over kinship type ($k$ =MOP in this instance), the reproductive output of an individual in year $t$ is a random variable $R(t)$, ${\mathbb{E}}[{R}_{i}({y}_{j})|{{\varvec{z}}}_{i}, {{\varvec{z}}}_{j}]$ is the expected reproductive output of female $i$ in the year of $j$’s birth, and ${\mathbb{E}}[{R}_{+}({y}_{j})|{{\varvec{z}}}_{j}]$ is the total expected reproductive output of all adult females in year ${y}_{j}$. Formulations are similar for other kinship types but require the re-expression of this ERRO principle in terms of case-specific covariates and parameters (see Sect. 3 of Bravington et al.^10^ for examples). The difficulty of re-expression depends on the biology and sampling of the species, with integrating uncertain age a challenging example.

The goal is to make inferences about abundance and demographic parameters, which requires a population dynamics model that evolves deterministically or stochastically depending on survival and birth rate parameters. The population dynamics model determines the elements needed to compute the kinship probabilities and inference on the parameters governing this model proceeds by maximizing the pseudo-log-likelihood

1 $$\ell \left({\varvec{\theta}}\right) ={\sum }_{k}{\sum }_{1\le \mathrm{i}\le \mathrm{j}\le n}[(1 -{ x}_{\mathit{ijk}})\mathrm{ log}(1 - {P}_{ijk}) + {x}_{ijk}\mathrm{log}({P}_{ijk})] ,$$

where ${x}_{ijk}$ is an observed binary indicator for whether individuals $i$ and $j$ are a match for kinship type $k$, which indexes over the different kinship types considered e.g., parent–offspring and half-siblings, and $i$ and $j$ index over the $n$ individuals. Equation (1) is a pseudo-likelihood because the pairwise comparisons are not strictly statistically independent e.g., because no animal can have more than one mother. However, pseudo-maximum-likelihood estimates are asymptotically unbiased, and variance estimates are accurate when sample sizes are small relative to the size of the population (see Sect. 4. of Bravington et al.^10^ for a discussion and Supplement of McDowell et al.^43^).

Inference can proceed by maximising Eq. (1) directly or be made more computationally efficient by factoring the likelihood into classes with the same covariate information, where the number of pairs in each class is a sum over Bernoulli random variables and thus follows a Binomial distribution.

### Female-focussed CKMR model

We initially investigate a female-focussed CKMR model because we were surer of the female-related biological assumptions, and it is simpler than the both-sex model. The following exposition serves as a template for the population dynamics model and kinship probabilities that are required for the extended model that includes both sexes.

The base population dynamics model describes the number of female adults ${N}_{{\female},t}\mathrm{ p}$ resent at an arbitrary unit of discrete-time *t*, taken to be years from here on, by the function

2 $${N}_{{\female},\mathrm{ t}}={N}_{{\female},{t}_{0}}\mathrm{exp}\left[{\rho }_{{\female}}\left(t-{t}_{0}\right)\right]$$

where ${N}_{{\female},{t}_{0}}$ is the adult female abundance at the initial year of modelling ${t}_{0}$, and ${\rho }_{{\female}}={\beta }_{{\female}}-{\delta }_{{\female}}$ is the female population rate of increase and balances the birth rate $\beta$ with the mortality rate $\delta$ assuming that the population is closed to immigration. In the CKMR model, $\beta$ is not explicitly estimated as $\rho$ is used in the population dynamics equations to compute ${N}_{{\female}, t}$ and $\delta$ in the kinship probability computation (see below). Importantly, ${N}_{{\female}}$ refers to the number of breeding adult females and ${\delta }_{{\female}}$ includes a rate of reproductive senescence alongside death i.e., the model cannot distinguish between these two concepts with just $\delta$. For females, we assume that reproductive senescence is not substantial, but this is an important consideration for males (see below). Therefore, for CIFF, the base case CKMR demographic model contains three parameters ${\varvec{\theta}}$ $={\left({N}_{{\female},{t}_{0}}, {\rho }_{{\female}}, {\delta }_{{\female}}\right)}^{{{\prime}}}$.

Given the demographic model, we require the kinship probabilities for POPs and HSPs, which link the genetic relationship to the demographic model. In the sampling exercises from which genetic samples for this study were obtained, the individual was released after tissue samples and measurements were taken. Furthermore, adults ages $\ge$ 36 months are considered unknown. Therefore, we require a form for the parent–offspring probabilities that can accommodate unknown adult age and encompass both the cases when the parent was sampled after the offspring and when the parent was sampled (potentially as a juvenile) before the offspring was sampled. POP probabilities can be split into MOPs and FOPs as sex is known.

Supplementary Note E details the derivation of the CIFF-specific CKMR probabilities. We state the probabilities for female parent comparisons only. For MOP comparisons, we wish to find the probability that individual $i$, sampled at time ${t}_{i}$, is the mother of juvenile *j*, born at time ${y}_{j}$. We assume that once a female reaches maturity, she has a fixed fecundity that is independent of covariates such as age. The probability that individual $i$ is $j$’s mother is

3 $${\mathbb{P}}({K}_{ij}= \mathrm{MOP} | {{\varvec{z}}}_{i},{{\varvec{z}}}_{j}) = \left\{\begin{array}{ll}\frac{\varphi ({y}_{j},{ t}_{i}) }{{N}_{{\female},{t}_{i}}}&\quad if\; {y}_{j} < {t}_{i} \\ \frac{\varphi ({t}_{i},{ y}_{j}) }{{N}_{{\female},{y}_{j}}}&\quad if\; {t}_{i} < {y}_{j},\end{array}\right.$$

where ${{\varvec{z}}}_{i}$ and ${{\varvec{z}}}_{j}$ are the vector of observed covariates for individual $i$ and $j,$ which include sample date for mother and juvenile, age of the juvenile to compute birth date and sex of the parent is implicit, $\varphi ({t}_{1},{t}_{2})$ is the survival function that specifies the survival rate of an individual between time ${t}_{1}$ and ${t}_{2}$ (${t}_{1}$< ${t}_{2}$) and is governed by the mortality rate *δ* via $\varphi ({t}_{1},{t}_{2}) =\mathrm{ exp}[-\delta ({t}_{2}- {t}_{1})].$ Equation (3) does not explicitly state that the mother was mature at the birth of the juvenile. In practice for CIFF, this constraint is enforced by removing, for the POP component of the model, adult-adult comparisons where the age of the potential offspring was greater than 36 months and comparisons where the older individual of the pair (earlier birth year) was caught as a juvenile and could not have been old enough to be an adult at the birth of the juvenile.

We note that the terms survival and mortality will both be used throughout and are to be treated as having a one-to-one correspondence via $\varphi$. The case where ${t}_{i}={y}_{j}$ is excluded as adults sampled during breeding season may not get the full opportunity to contribute reproductively that year or the juvenile may be sampled with the mother. Importantly, these equations only rely on the birth year of the juvenile and the sampling year of the adult. The second case of Eq. (3) is specific to the mark-recapture sampling scenario where the mother can be caught before the juvenile is born.

Bravington et al.^10^ details the HSP probability calculation (see Sect. 3.2 of Bravington et al.^10^). Briefly, we take some unknown but fixed individual *m* as the true mother of $i$. Thus, we know *m* was alive and mature at ${y}_{i}$, which is analogous to *m* having been sampled at ${y}_{i}$. This implies that the probability that $i$ and *j* form a maternal HSP (MHSP) is simply the probability that *m* is the mother of *j*, with ${t}_{m}={y}_{i}<{y}_{j}$. Therefore, the MHSP probability is given by

4 $${\mathbb{P}}({K}_{ij}= \mathrm{MHSP} | {{\varvec{z}}}_{i},{{\varvec{z}}}_{j})=\frac{\varphi ({y}_{i},{ y}_{j}) }{{N}_{{\female},{y}_{j}}}$$

Equation (4) assumes that individual $i$ must be born first i.e., ${y}_{i}<{y}_{j}$ such that same-cohort cases are excluded.

To implement the female-focussed model we need to differentiate the sex of the shared parent for putative siblings. To accomplish this, we used mtDNA sequencing. If haplotypes for the mtDNA region sequenced are shared, then this is evidence for the pair being maternally linked. However, if there are mtDNA haplotypes of substantial frequency (specifically less haplotypes than there are females) in the population, half siblings may share a father but maintain a shared haplotype because each individual’s mother by chance could have the same haplotype. Therefore, to adequately incorporate the potentially increased presence of FSPs due to roost and mate fidelity and dominant males along with uncertainty in mtDNA haplotype sharing due to chance haplotype sharing, we derived sibling kinship probabilities that describe the three sibling cases. That is, the pair shares both parents, the pair shares just a mother, or the pair shares just a father (see Supplementary Note F for details). The sibling probability calculation is described in Supplementary Eq. (S5).

Fitting the female-focussed model is not straightforward because the sibling probability and the inclusion of uncertainty in haplotype sharing require male dynamics and kinship probabilities to be calculated. Supplementary Note G details how this can be achieved without male components through a few added assumptions. The final paragraphs of the “Methods” section in Supplementary Note G provide the main components for reconstructing the estimation procedure for the female-focussed model and incorporate some of the concepts detailed in the extended models below.

### Extended CKMR models

The extended models include male populations dynamics and kin and attempt to model male reproductive skew and mate persistence. For CIFF, we observed substantial numbers of intra-cohort HSPs, which should be rare under random mating given the constraint that females can only have one pup per year. Their presence implies that these intra-cohort HSPs are paternally derived and are a result of male reproductive skew. Furthermore, if males repeatedly mate with the same set of females, dominate a breeding location year-on-year, or both, then full sibling pairs (FSPs) may be present in the kinship analyses, which was the case for CIFF. We present an initial base model that includes these concepts and explore extra parameters that are biologically plausible.

The population dynamics for total adult abundance is modelled as

5 $${N}_{t}={N}_{{\female},{t}_{0}}\mathrm{exp}\left[{\rho }_{{\female},{\male}}\left(t-{t}_{0}\right)\right] + {N}_{{\male},{t}_{0}}\mathrm{exp}\left[{\rho }_{{\female},{\male}}\left(t-{t}_{0}\right)\right]$$

$\mathrm{where} {N}_{{\female},{t}_{0}}$ and ${N}_{{\male},{t}_{0}}$ are the adult female and adult male abundance at the initial year of modelling *t*_0_, and ${\rho }_{{\female},{\male}}$ is now a common trend parameter for adult females and males. In the extended model, the MOP and FOP probabilities are computed as per Eq. (3) with the denominator replaced with the corresponding adult male abundance value for paternal kin probabilities. As for the female-focussed model, the MHSP and PHSP calculations are included in a broader 'sibling pair' probability calculation that include FSPs (detailed in Eq. (5) of Supplementary Note F).

Briefly, the sibling probability is computed by summing over three demographic probabilities FSP, MHSP, and PHSP. We let *M* be the event that pair ($i$*,* *j*) share a mother and *P* the event that pair ($i$*,* *j*) share a father. We propose the following model for the FSP probability that incorporates the potential for mate persistence across breeding seasons

6 $${\mathbb{P}}\left(M\cap P|{{\varvec{z}}}_{i},{{\varvec{z}}}_{j}\right)={\mathbb{P}}\left(P|{{\varvec{z}}}_{i},{{\varvec{z}}}_{j}\right)\times {\mathrm{logit}}^{-1}\left\{\alpha + \mathrm{logit}\left[{\mathbb{P}}\left(M|{{\varvec{z}}}_{i},{{\varvec{z}}}_{j}\right)\right]\right\},$$

where the parameter *α* models mate persistence and ${\mathbb{P}}(P|{{\varvec{z}}}_{i},{{\varvec{z}}}_{j})$ and ${\mathbb{P}}(M|{{\varvec{z}}}_{i},{{\varvec{z}}}_{j})$ are computed from Eq. (4) i.e., are the unadjusted maternal and paternal half-sibling probabilities, and Eq. (4) is computed such that ${y}_{i}<{y}_{j}$ i.e., same-cohort cases are excluded for sibling probabilities and comparisons. For Eq. (6) to be non-zero the sibling pair’s father and mother must survive and have a non-zero breeding probability, which is zero if the maternal probability is zero (e.g., intra-cohort FSP). This calculation requires the paternal half-sibling probability, and, in turn, a male abundance parameter.

To incorporate male reproductive skew, we make the important distinction between the total set of adult males and the breeding set of males in the population. We propose that the paternal POP component of the model estimates the total set of males while the paternal sibling component estimates the breeding set. The intuition for this is that the comparisons made for the POP component of the model include all sampled males as potential parents, which includes those that are not contributing to reproduction. The sibling component of the model tracks the unobserved shared parent of the two siblings and is therefore only providing information on the breeding males. Therefore, the difference between these two components of the model allows for an estimate of the proportional of total males that are breeding.

To make this distinction in the model, we incorporate breeding male population size in the computation via ${N}_{{\male},{t}_{0}}\times \pi$, where $\pi$ lies in (0*,* 1) and models loosely the proportion of total males at time ${t}_{0}$ contributing to reproduction. The population rate of increase and mortality parameters are initially shared between the female and male components of the model. The parameter set to be estimated for the base model is now

$${\varvec{\theta}}={\left({N}_{{\female},{t}_{0}}, { N}_{{\male},{t}_{0}}, {\rho }_{{\female},{\male}}, { \delta }_{{\female},{\male}, } \alpha , \pi \right)}{^\prime}.$$

Initial investigations indicated that mortality may be estimated with poor accuracy given the available data. A prior MR study^42^ for CIFF had estimated survival and we, therefore, explored model fit when the mortality parameter was constrained through a prior that has a Gaussian distribution with expectation ${\mu }_{{\delta }_{{\female},{\male }}}= 0.443$ and ${\sigma }_{{\delta }_{{\female},{\male }}}^{2}= 0.206$. These values corresponded to the transformed weighted (recapture sample size per year) average of the adult female survival values of $\mu =0.643$ and SE = 0.134 reported in Todd^42^.

We propose that the mortality rate of the breeding set of males includes not only death but an extra rate of dominant male turnover. We investigated model fit with an extra mortality parameter ${\delta }_{{\male}*}$ that is included in the paternal sibling calculations i.e., the paternal sibling components of Supplementary Eq. (S5) are computed with their own mortality parameter and all other kinship components (MOP, POP and MHSP) are computed with a common mortality parameter. Overall, we explore parameter estimates and model fit for four extended models. The base model is the simplest with no prior on mortality and no breeding male mortality parameter. The other three models complete the two-by-two table of common mortality parameter prior and breeding male mortality parameter.

### Parameter estimation and model validation

The pseudo-likelihood function proposed in Bravington et al.^10^ and summarised in Eq. (1) is used to approximate the full likelihood function and assumes independence between all kin-pairs and involves only the marginal probabilities detailed in Eqs. (3)–(4).

For CIFF, many pairwise comparisons in the computation of Eq. (1) have the same kinship covariates ${({\varvec{z}}}_{i},{ {\varvec{z}}}_{j})$(e.g., birth cohort, sampling year). To improve computational efficiency, we aggregate comparisons into classes with equal kinship probabilities. We implemented a covariate-class Binomial log pseudo-likelihood version of Eq. (1) (see Supplementary Eq. S12). Estimates of ${\varvec{\theta}}$ are obtained via maximising Supplementary Eq. (S12) using the nlminb function in the R programming language^55,56^. The gradient is supplied to the optimisation routine and is computed using automatic differentiation via the Template Model Builder (TMB) package^57^, which integrates C++ with R. Automatic differentiation in TMB facilitates the estimation of parameter uncertainty, which is computed for all model parameters. Hessian-based parameter standard errors were obtained using TMB algorithmic differentiation functions and interval estimates were reported by appropriate transformation. Hessian-based standard errors are appropriate for CKMR provided approximate independence holds (see Sect. 4. of Bravington et al.^10^ and Supplement of McDowell et al.^43^). One heuristic for assessing approximate independence outlined in Bravington et al.^10^ inspects the ratio of kin-triads (within which the pairwise comparisons are clearly not independent) to kin-pairs. If this rate is low, say on the order of the inverse of the root sample size, then independence is a reasonable assumption. To investigate this assumption, we inspect the ratio of kin clusters to kin found for CIFF. For further checks on symmetry of likelihood around MLE estimates, profile likelihood confidence intervals were also computed using the tmbprofile and cofint.tmbprofile functions in TMB, which implements the intervals based on Wilks' theorem (Sect. 13.8 of Wilks^58^).

The score function of the log pseudo-likelihood at the estimates reported from TMB was evaluated to ensure values were near zero. Model fit was evaluated by comparing observed versus expected kin for each of the modelled kinship types (MOP, FOP, MHSP, PHSP, FSP). The expected number of kin is computed as the number of combinations multiplied by the kinship probabilities for each kinship type computed under the maximum likelihood estimates (MLE) of ${\varvec{\theta}}$. Inadequate model fit was evaluated by computing whether the observed kin count was within the 95% interval of the Poisson distribution with rate parameter equal to the expected number of kin. The Poisson was used for ease of reporting and reference, where the interval can simply be computed under the Poisson distribution using the reported expected number of kin, whereas the probability and number of comparisons for each cell would be required for the Binomial calculation. The Poisson distribution is a very good approximation to the Binomial when the number of comparisons is large and the probabilities of kinship small, which is the case for CKMR. Goodness of fit measures were also computed for the extended models by summing over the squared observed minus expected (normalised by expected value) kin differences for each kin category.

To visualise the variability in trend given the uncertainty in parameter estimates from each model we sampled ten potential trends using the sampling importance resampling (SIR) algorithm^59^. Briefly, we drew 1000 replicates from the multivariate normal distribution (MVN) with expectation at the MLE estimates and the estimated covariance matrix of the parameter estimates reported from TMB. For each draw, we computed the CKMR likelihood value at that draw and divided it by the MVN density value at that draw, which becomes the weight for this draw. From these 1000 draws (and weights) we took a weighted random sample of 10 sets of parameter estimates. These parameter sets generate trend sets that are representative of the potential trends in abundance given the uncertainty in the distribution of parameter estimates.

### Ethics declarations

The authors have complied with ARRIVE guidelines.

## Supplementary Information

Supplementary Information 1.

Supplementary Information 2.

## Data Availability

Raw and processed genotype data, mitochondrial DNA data along with Template Model Builder C++ code, R code, and summary results generated are available at CSIRO's Data Access Portal permanent repository at 10.25919/2k26-t773. Results of the present study were generated from R version 4.1.2.

## References

[1] Krebs CJ (1985). Ecology; the experimental analysis of distribution and abundance.

[2] MacKenzie, D. I. *et al. Occupancy estimation and modeling: inferring patterns and dynamics of species occurrence*. (Elsevier, 2017).

[3] Anderson R, Gordon DM, CraWley M, Hassell MP (1982). Variability in the abundance of animal and plant species. Nature.

[4] Lande R (1988). Genetics and demography in biological conservation. Science.

[5] Begon, M. & Townsend, C. R. *Ecology: from individuals to ecosystems*. (John Wiley & Sons, 2020).

[6] Hilborn R (2020). Effective fisheries management instrumental in improving fish stock status. Proc. Natl. Acad. Sci..

[7] Jolly GM (1965). Explicit estimates from capture-recapture data with both death and immigration-stochastic model. Biometrika.

[8] Seber GA (1965). A note on the multiple-recapture census. Biometrika.

[9] Luikart G, Ryman N, Tallmon DA, Schwartz MK, Allendorf FW (2010). Estimation of census and effective population sizes: the increasing usefulness of DNA-based approaches. Conserv. Genet..

[10] Bravington MV, Skaug HJ, Anderson EC (2016). Close-kin mark-recapture. Stat. Sci..

[11] Hillary RM (2018). Genetic relatedness reveals total population size of white sharks in eastern Australia and New Zealand. Sci. Rep..

[12] Waples RS, Feutry P (2022). Close-kin methods to estimate census size and effective population size. Fish Fish..

[13] Beeton, B. *et al.* Final Report Christmas Island Expert Working Group to Minister for the Environment. *Heritage and the Arts* (2010).

[14] Zichy-Woinarski, J. C., Burbidge, A. & Harrison, P. *The action plan for Australian mammals 2012*. (CSIRO publishing, 2014).

[15] James, D. J., Dale, G. J., Retallick, K. & Orchard, K. Christmas Island flying-fox *Pteropus natalis* Thomas 1887: an assessment of conservation status and threats. *Christmas Island National Park, Commonwealth of Australia* 55 (2007).

[16] Woinarski JC (2014). An island-wide monitoring program demonstrates decline in reporting rate for the Christmas Island flying-fox Pteropus melanotus natalis. Acta Chiropterol..

[17] Director of National Parks. *Status of the Christmas Island flying fox: results from the 2016 island-wide monitoring program.* (2016).

[18] Dorrestein A, Todd CM, Westcott DA, Martin JM, Welbergen JA (2019). Impacts of an invasive ant species on roosting behavior of an island endemic flying-fox. Biotropica.

[19] Pulscher LA (2021). Evidence of chronic cadmium exposure identified in the critically endangered Christmas Island flying-fox (Pteropus natalis). Sci. Total Environ..

[20] Tidemann, C. R. A study of the status, habitat requirements and management of the two species of bat on Christmas Island (Indian Ocean): final report. *Zoology Department, Australian National University, Canberra, Australian Capital Territory, Australia* (1985).

[21] Threatened Species Scientific Committee. Commonwealth conservation advice for Pteropus natalis (Christmas Island flying-fox). *Department of the Environment* 1–15 (2014).

[22] Geyle HM (2018). Quantifying extinction risk and forecasting the number of impending Australian bird and mammal extinctions. Pac. Conserv. Biol..

[23] Director of National Parks. *Status Report–September 2014 Monitoring, Research and Management of the Christmas Island flying-fox (Pteropus melanotus natalis)*. (2014).

[24] Todd CM (2022). Body-size dependent foraging strategies in the Christmas Island flying-fox: Implications for seed and pollen dispersal within a threatened island ecosystem. Mov. Ecol..

[25] Skaug HJ (2001). Allele-sharing methods for estimation of population size. Biometrics.

[26] Nichols JD, Hines JE, Pollock KH (1984). Effects of permanent trap response in capture probability on Jolly-Seber capture-recapture model estimates. J. Wildl. Manag..

[27] Wegge, P., Pokheral, C. P. & Jnawali, S. R. Effects of trapping effort and trap shyness on estimates of tiger abundance from camera trap studies. in *Animal Conservation Forum* vol. 7, pp. 251–256 (Cambridge University Press, 2004).

[28] Gallagher AJ, Staaterman ER, Cooke SJ, Hammerschlag N (2017). Behavioural responses to fisheries capture among sharks caught using experimental fishery gear. Can. J. Fish. Aquat. Sci..

[29] Feutry P (2017). Inferring contemporary and historical genetic connectivity from juveniles. Mol. Ecol..

[30] Conn, P. B., Bravington, M. V., Baylis, S. & Ver Hoef, J. M. Robustness of close‐kin mark–recapture estimators to dispersal limitation and spatially varying sampling probabilities. *Ecol. Evol.* **10**, 5558–5569 (2020). 10.1002/ece3.6296 PMC7319163 32607174

[31] Bravington MV, Grewe PM, Davies CR (2016). Absolute abundance of southern bluefin tuna estimated by close-kin mark-recapture. Nat. Commun..

[32] Wacker S (2021). Considering sampling bias in close-kin mark–recapture abundance estimates of Atlantic salmon. Ecol. Evol..

[33] Bradford, R. *et al. A close-kin mark-recapture estimate of the population size and trend of east coast grey nurse shark. Report to the National Environmental Science Program, Marine Biodiversity Hub*. (CSIRO Oceans & Atmosphere, Hobart, Tasmania, 2018).

[34] Bravington, M. *et al.* Close-Kin Mark-Recapture population size estimate of *Glyphis garricki* in the Northern Territory. *Report to the National Environmental Science Program, Marine Biodiversity Hub. CSIRO Oceans & Atmosphere, Hobart* (2019).

[35] Ruzzante DE (2019). Validation of close-kin mark–recapture (CKMR) methods for estimating population abundance. Methods Ecol. Evol..

[36] Sansaloni, C. *et al.* Diversity Arrays Technology (DArT) and next-generation sequencing combined: genome-wide, high throughput, highly informative genotyping for molecular breeding of Eucalyptus. in *BMC proceedings* vol. 5 1–2 (BioMed Central, 2011).

[37] Kilian, A. *et al.* Diversity arrays technology: a generic genome profiling technology on open platforms. in *Data production and analysis in population genomics* 67–89 (Springer, 2012). 10.1007/978-1-61779-870-2_5 22665276

[38] Melville J (2017). Identifying hybridization and admixture using SNPs: Application of the DArTseq platform in phylogeographic research on vertebrates. R. Soc. Open Sci..

[39] Phalen DN (2017). Genetic diversity and phylogeny of the Christmas Island flying fox (*Pteropus melanotus natalis*). J. Mammal..

[40] Todd CM, Westcott DA, Rose K, Martin JM, Welbergen JA (2018). Slow growth and delayed maturation in a critically endangered insular flying fox (*Pteropus natalis*). J. Mammal..

[41] Bravington, M. V., Miller, D. L. & Baylis, S. M. Kinference: pair wise kin-finding for close-kin mark-recapture. R package version 0.0.80 (2021).

[42] Todd, C. M. The ecology and conservation of the Christmas Island flying-fox (*Pteropus natalis*). (Western Sydney University, 2020).

[43] McDowell JR (2022). Low levels of sibship encourage use of larvae in western Atlantic bluefin tuna abundance estimation by close-kin mark-recapture. Sci. Rep..

[44] Corbett, L., Crome, F. & Richards, G. Fauna survey of mine lease applications and national park reference areas, Christmas Island, August 2002. Appendix G. *Phosphate Resources Limited (eds.)* (2003).

[45] McCracken, G. F. & Wilkinson, G. S. Bat mating systems. in *Reproductive biology of bats* 321–362 (Elsevier, 2000).

[46] Carroll JB, Mace GM (1988). Population management of the Rodrigues fruit bat. Int. Zoo Yearbook.

[47] Rossiter SJ, Ransome RD, Faulkes CG, Le Comber SC, Jones G (2005). Mate fidelity and intra-lineage polygyny in greater horseshoe bats. Nature.

[48] Welbergen, J. A. The social organisation of the grey-headed flying-fox, Pteropus poliocephalus. (University of Cambridge, 2005).

[49] Feutry P (2020). One panel to rule them all: DArTcap genotyping for population structure, historical demography, and kinship analyses, and its application to a threatened shark. Mol. Ecol. Resour..

[50] Divljan A, Parry-Jones K, Wardle GM (2006). Age determination in the grey-headed flying fox. J. Wildl. Manag..

[51] Mayne B (2021). Nonlethal age estimation of three threatened fish species using DNA methylation: Australian lungfish, Murray cod and Mary River cod. Mol. Ecol. Resour..

[52] Lu, A. T. *et al.* Universal DNA methylation age across mammalian tissues. *BioRxiv* 2021.01. 18.426733 (2021).

[53] Patterson, T. A. *et al.* Rapid assessment of adult abundance and demographic connectivity from juvenile kin pairs in a critically endangered species. *Sci. Adv.* **8**, eadd1679 (2022). 10.1126/sciadv.add1679 PMC9770943 36542711

[54] Sikes RS, Gannon WL (2011). Guidelines of the American Society of Mammalogists for the use of wild mammals in research. J. Mammal..

[55] Gay DM (1990). Usage summary for selected optimization routines. Comput. Sci. Tech. Rep..

[56] R. Core Team. R: A language and environment for statistical computing. (2021).

[57] Kristensen K, Nielsen A, Berg CW, Skaug H, Bell BM (2016). TMB: Automatic differentiation and laplace approximation. J. Stat. Soft..

[58] Wilks, S. S. *Mathematical statistics* (1964).

[59] Rubin DB (1987). The calculation of posterior distributions by data augmentation: Comment: A noniterative sampling/importance resampling alternative to the data augmentation algorithm for creating a few imputations when fractions of missing information are modest: The SIR algorithm. J. Am. Stat. Assoc..

