# Supplementary material for: Close-kin mark-recapture informs critically endangered terrestrial mammal status
Source: Sci Rep. 2023 Aug 2;13:12512. doi: 10.1038/s41598-023-38639-z (PMC10397353; doi:10.1038/s41598-023-38639-z)
Supplement: Supplementary file 1 — Supplementary Information 1. [file 41598_2023_38639_MOESM1_ESM.pdf]

Close-kin mark-recapture informs critically endangered terrestrial mammal status - Supplementary Information

Luke R. Lloyd-Jones[1], Mark V. Bravington[2], Kyle N. Armstrong[3], Emma Lawrence[1], Pierre Feutry[4], Christopher M. Todd[5], Annabel Dorrestein[5], Justin A. Welbergen[5], John M. Martin[6], Karrie Rose[7], Jane Hall[7], David N. Phalen[8], Isabel Peters[9], Shane M. Baylis[4], Nicholas A. Macgregor[10,11], and David A. Westcott[12]

[1]Commonwealth Scientific and Industrial Research Organisation, Data61, Brisbane, Queensland 4072, Australia
[2]Commonwealth Scientific and Industrial Research Organisation, Data61, Hobart, Tasmania, 7000, Australia
[3]Environment Institute, University of Adelaide, North Terrace, Adelaide, South Australia, 5005, Australia
[4]Commonwealth Scientific and Industrial Research Organisation, Oceans and Atmosphere, Hobart, Tasmania, 7000, Australia
[5]The Hawkesbury Institute for the Environment, Western Sydney University, Richmond, New South Wales, Australia
[6]Royal Botanic Gardens and Domain Trust, Sydney, New South Wales, 2000, Australia
[7]Australian Registry of Wildlife Health, Taronga Conservation Society Australia, Bradleys Head Road, Mosman, New South Wales, 2088, Australia
[8]Sydney School of Veterinary Science, Faculty of Science, University of Sydney, Sydney, New South Wales, 2006, Australia
[9]School of Mathematics and Physics, University of Queensland, Brisbane, Queensland, 4072, Australia
[10]Parks Australia, Canberra, Australian Capital Territory, 2601, Australia
[11]Durrell Institute of Conservation and Ecology (DICE), School of Anthropology and Conservation, University of Kent, Canterbury, Kent CT2 7NR, UK
[12]Commonwealth Scientific and Industrial Research Organisation, Land and Water, Atherton, Queensland, 4883, Australia

Contents

Supplemental Figures and Tables **3**

Supplementary Notes **10**

A Genotyping and quality control . . . 10

B Half-sibling pair detection . . . 15

C Aunt/Uncle-Niece/Nephew kin pair rate simulation study . . . 17

D Mitochondrial DNA sequencing and quality control . . . 19

E Derivation of kin probabilities . . . 20

F Derivation of sibling probability and Binomial class log pseudo-likelihood 26

G Reduced female-only model . . . 31

H Sensitivity of results over alternate models . . . 38

Supplemental Figures and Tables

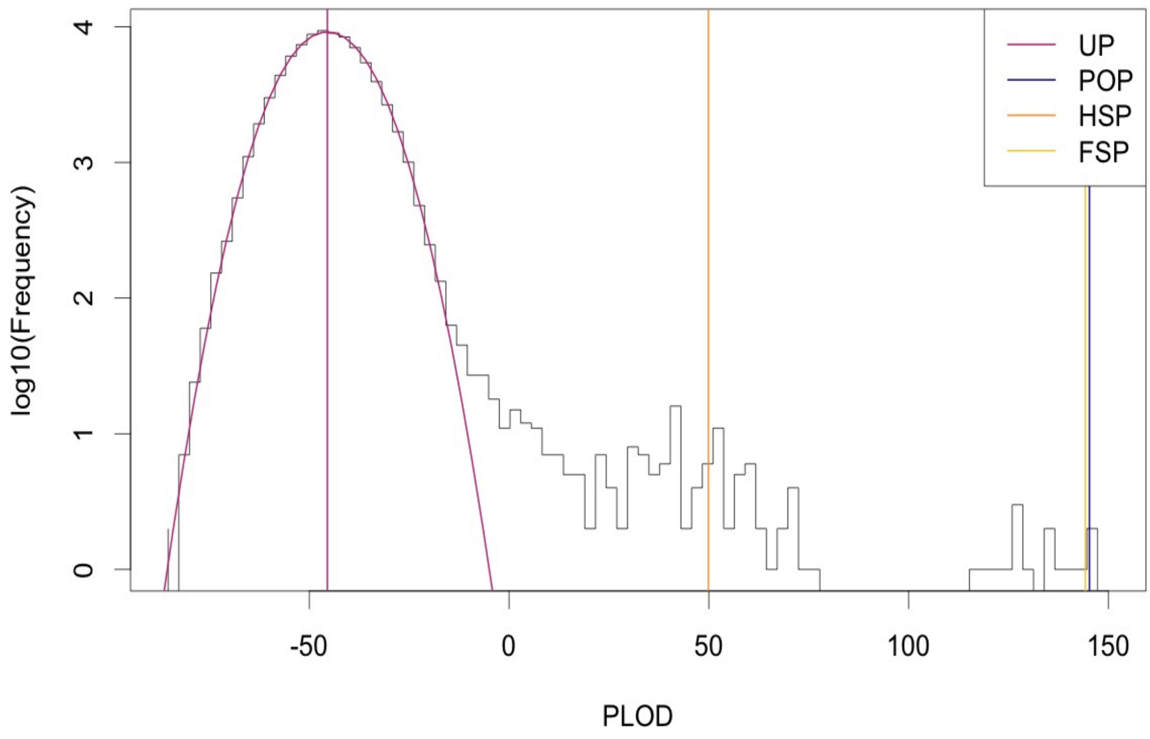

Figure S1 Observed log-frequencies of PLOD scores for all pairwise comparisons. The log number of pairs for a binned PLOD value is displayed by the black histogram bars. The large peak at the left of the plot indicates the distribution of PLODs for unrelated pairs (UP), and the purple curve and vertical line indicate the expected distribution of PLODs and mean for unrelated pairs respectively. At approximately PLOD = 50, there is a peak in the observed data where we expect (orange vertical line) the set of half-sibling pairs (HSP) PLODs to peak. The right of the plot shows vertical lines where the expected peaks for full-sibling pairs (FSP) and parent-offspring pairs (POP). There is a close alignment between the theoretical mean and the variance of the UP distribution (-45.5 and 85.7) and the empirical mean and variance (-45.5 and 93.1), which is further evidence that the genotype set is adequate for kin detection.

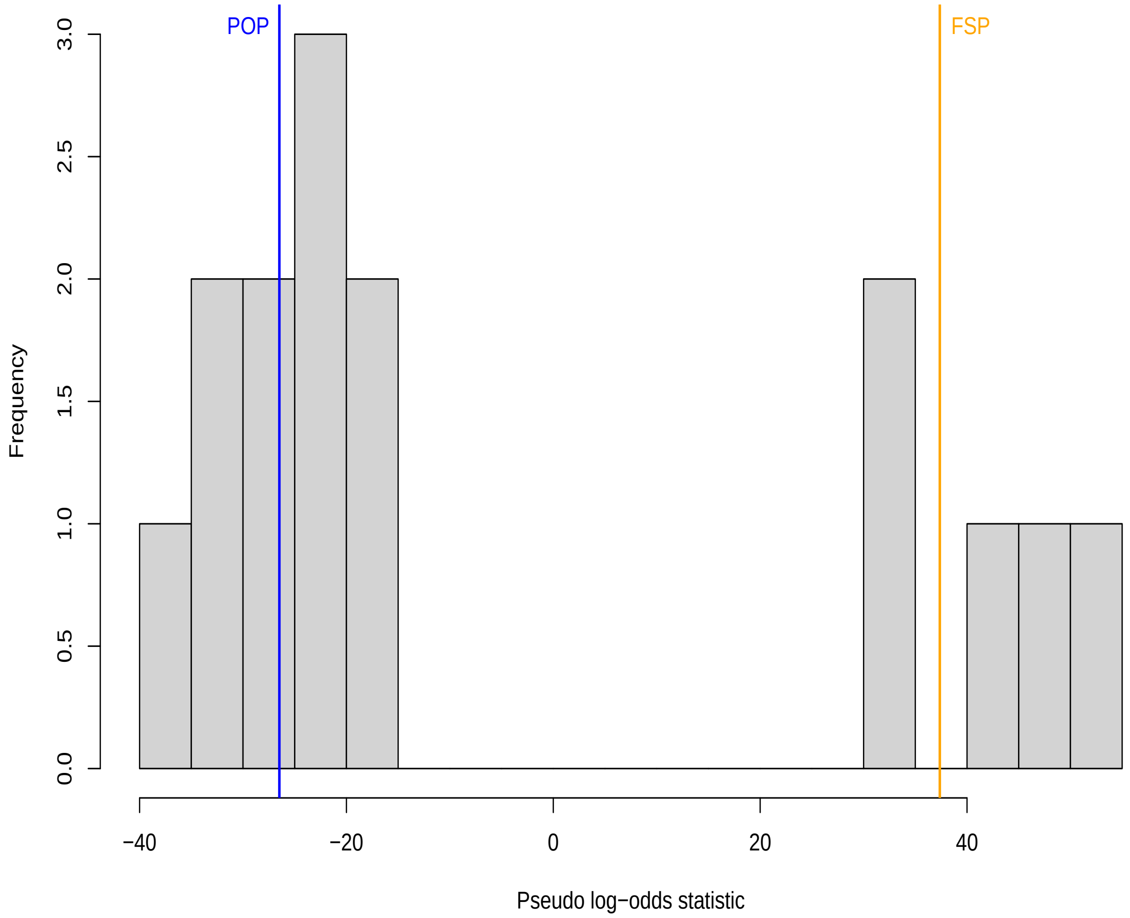

Figure S2 Observed frequencies of POP/FSP-specific PLOD scores for pairs showing evidence of being first-degree relatives. The histogram shows the discrimination of parent-offspring pairs (POPs) from full-sibling pairs (FSPs) (pairs with HSP/UP PLOD > 100 in Supplementary Figure S1) using the PLOD statistic tuned for this discrimination. The blue and orange vertical lines show the expected values of these statistics for each kinship type.

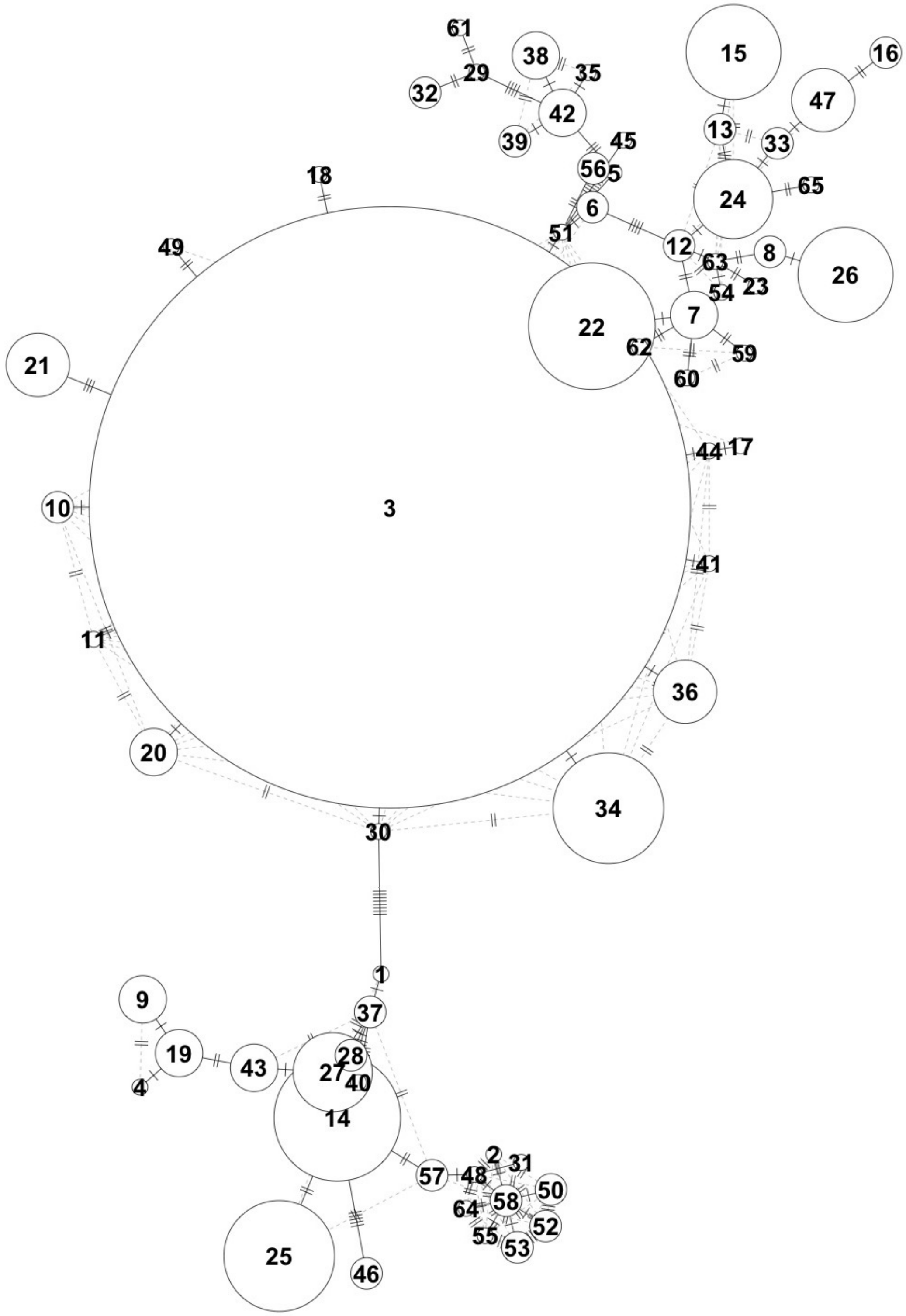

Figure S3 Haplotype network constructed using R package pegas[1], incorporating 187 CIFF mitochondrial DNA sequences. The size of the circle indicates the frequency of a given haplotype and cross lines separating haplotypes represent mutational steps between haplotypes. Numbers label the haplotypes and correspond to those reported in Supplementary Table S2.

	Juvenile	Sub-adult	Adult
Female proportion	0.384	0.339	0.371

Table S1 Proportion of female CIFFs in each age class for the total number of CIFF samples ($N = 728$) with wing-clip tissue.

Table S2 Separate comma-separated Excel spreadsheet file with all detected kin pairs and necessary covariate information with PLOD value $\geqslant 32$.

A MOP combination matrix

	2015	2016	2017	2018	2019
2013	67	180	138	69	460
2014	54	144	108	54	360
2015	0	840	628	315	2100
2016	273	0	546	273	1812
2017	288	792	0	216	1440
2018	594	1122	264	0	660

B F/H SP combination matrix

	2013	2014	2015	2016	2017	2018
2013	253	414	2438	2070	1656	759
2014	0	153	1908	1620	1296	594
2015	0	0	5565	9540	7632	3498
2016	0	0	0	4005	6480	2970
2017	0	0	0	0	2556	2376
2018	0	0	0	0	0	528

C MOP observed kin matrix

	2015	2016	2017	2018	2019
2013	0	0	0	0	0
2014	0	0	0	0	0
2015	0	0	0	0	0
2016	0	0	0	0	0
2017	0	0	0	0	0
2018	1	0	0	0	0

D F/H SP observed kin matrix

	2013	2014	2015	2016	2017	2018
2013	1	0	3	0	0	1
2014	0	0	2	1	2	2
2015	0	0	7	8	9	3
2016	0	0	0	9	9	5
2017	0	0	0	0	1	3
2018	0	0	0	0	0	2

Table S3 Maternal close-kin matrices for parent-offspring pairs and all full/half-siblings. Combination matrices detail the number of valid pairwise comparisons made to detect kin for each kin type and birth year/sampling year covariates. Kin matrices detail the number of kin detected in the Kinference analysis. For MOP matrices A) and C) the rows represent the birth year of the juvenile and the columns the sampling year of the mother. For F/MH SP matrices B) and D) the rows represent the birth year of the older juvenile and the columns the birth year of the younger juvenile. Diagonal elements of the MOP combination matrices are absent to remove the possibility of juveniles being sampled with the mother and the influence of sampling on reproduction in that year. Diagonal elements of the sibling matrix are presented to show the extent of intra-cohort pairs but are not used in the CKMR analysis.

A FOP combination matrix

	2015	2016	2017	2018	2019
2013	113	744	368	92	713
2014	90	587	288	72	558
2015	0	3465	1675	420	3255
2016	455	0	1456	364	2804
2017	504	2736	0	288	2232
2018	924	2475	561	0	1023

B FOP observed kin matrix

	2015	2016	2017	2018	2019
2013	0	0	0	0	0
2014	0	1	0	0	0
2015	0	1	0	0	0
2016	1	0	0	0	0
2017	0	1	0	0	0
2018	0	1	0	0	0

Table S4 Paternal close-kin matrices for parent-offspring pairs. Combination matrices detail the number of valid pairwise comparisons made to detect kin for each kin type and birth year/sampling year covariates. Kin matrices detail the number of kin detected in the Kinference analysis. For paternal parent-offspring pair (POP) matrices A) and C) the rows represent the birth year of the juvenile and the columns the sampling year of the father. Diagonal elements of the POP combination matrices are absent to remove the possibility of juveniles being sampled with the mother and the influence of sampling on reproduction in that year.

Supplementary Notes

A Genotyping and quality control

To detect kin pairs, we performed single nucleotide polymorphism (SNP) genotyping for each available CIFF individual. Wing-membrane biopsies were assessed with 693 individuals having available and substantial tissue for genetic analyses. DNA extraction and quality control were performed by Diversity Arrays Technology (DArT P/L, Canberra). Quality control at DArT removed 197 individuals with poor extraction and complexity reduction preparation, which was hypothesised to be a result of either tissue quality, microbial contamination, or both. Samples passing quality control were genotyped using DArTseqTM[2–4]. DArTseq combines organism-tailored complexity reduction methods and next-generation sequencing (NGS) platforms. DArTseq is optimized for each organism by selecting the complexity reduction method that allows for the detection of a high number of informative SNPs across the genome while minimising sequencing costs. Multiple methods of complexity reduction were tested and the PstI-SphI and PstI-HpaII reduction enzyme sets[3] were selected as the most appropriate complexity reduction method, both in terms of the size of the representation and the fraction of the genome selected for assays. For SNP calling, all tags from all libraries included in the analysis were clustered using a DArT proprietary C++ algorithm (DArTsoft14), followed by parsing of the clusters into separate SNP loci using a range of technical parameters, especially the balance of read counts for the allelic pairs. In addition, approximately 30% of the samples, referred to as technical replicates, were processed multiple times from library preparation to allelic calls and scoring consistency was used as the main selection criterion for high-quality/low error rate markers. Calling quality was assured by high average read depth per locus. The dataset used for population analysis from the DArTsoft14 pipeline consisted of 75 base pair (bp) fragments containing one or more SNPs. We removed one of each pair of duplicates favouring higher genotype call rate individuals in the pair. Post DArT Pty Ltd quality control and duplicate removal, the DArTseq analysis reported 448 (197 lost to poor DNA quality and 45 to duplication) individuals and 3,933 loci for the

PstI-SphI complexity reduction.

For the DArT genotype calls from the PstI-SphI analysis, we performed further quality control using an internal R programming language package Kinference[5]. We assumed a four-way coding of the genotypes, that is, at any given locus the possible genotypes are AA0, AB, BB0, and 00, where A and B are the SNP alleles and 0 denotes a null allele. Conceptually in the computations of the Kinference R package, there are no missing values. Nulls are assumed to represent real heritable allelic variants see Hillary et al.[6] for a more detailed discussion of this encoding and allele frequency estimation. The ABO genotypes used to encode the genotype value are expected to follow HWE proportions, and we test that assumption in the HWE calculations and other quality control steps. Low-frequency SNPs are less useful for kin finding because key statistics computed from them are noisy and genotype calls are more likely to contain influential errors. Therefore, SNPs with minor allele frequencies (MAFs) less than 0.02 were removed (1,038 SNPs removed). SNPs with genotype null rates greater than 0.1 were also removed (757 SNPs removed). Although Kinference can model the frequency of null alleles, experimentation with the maintenance of variants with null rates greater than 0.1 biased the primary kin-finding statistics, which may indicate inadequate average sequencing depth to reliably model and call null alleles. Initial checks of the genotype frequencies against Hardy-Weinberg equilibrium (HWE) expectations for the four-way genotype coding showed some variants deviating from expectation. Variants with a HWE chi-squared test p-value $< 1 \times 10^{-3}$ were removed (53 SNPs removed) (see Figure S4 A)). Loci with low reproducibility (< 0.95) score, which summarises the proportion of identical genotypes calculated from technical replicates routinely included by DArT P/L for routine quality control indicating loci subject to higher genotyping error rates, were removed (64 loci removed, see Figure S4 B)).

Initial quality control for kin detection required the removal of individuals that showed evidence of atypical genotypes compared to the population using a likelihood score (computed from observed versus expected genotypes using the `ilglk_geno` function in the Kinference R package) summed over all loci for an individual. We compared the ob-

served distribution of likelihood scores with the predicted theoretical distribution computed from population allele frequencies. Individuals whose likelihood score exceeded the tails of the predicted theoretical distribution were removed. Outlier individuals in this diagnostic test could have degraded or contaminated samples. This quality control step is performed in stages with initial lower extremes removed and then allele frequencies recomputed. For CIFF on only filtering on the lower tail was required as is typically the case for this statistic (see Figure S5 panels A) to C))

Further diagnostic checks for deviations from expected heterozygosity summed over loci for an individual were performed using the `hetzminoo_fancy` function in Kinference. We compared the observed distribution of heterozygosity scores with the predicted theoretical distribution computed from population allele frequencies and individuals that exceeded the tails of the predicted theoretical distribution were removed (see Figure S5 panels D) to E)). These checks provided further quality assurance for whether samples are likely to be contaminated or degraded. Following these checks, 409 individuals and 2,021 variants were retained for kinship analysis.

A

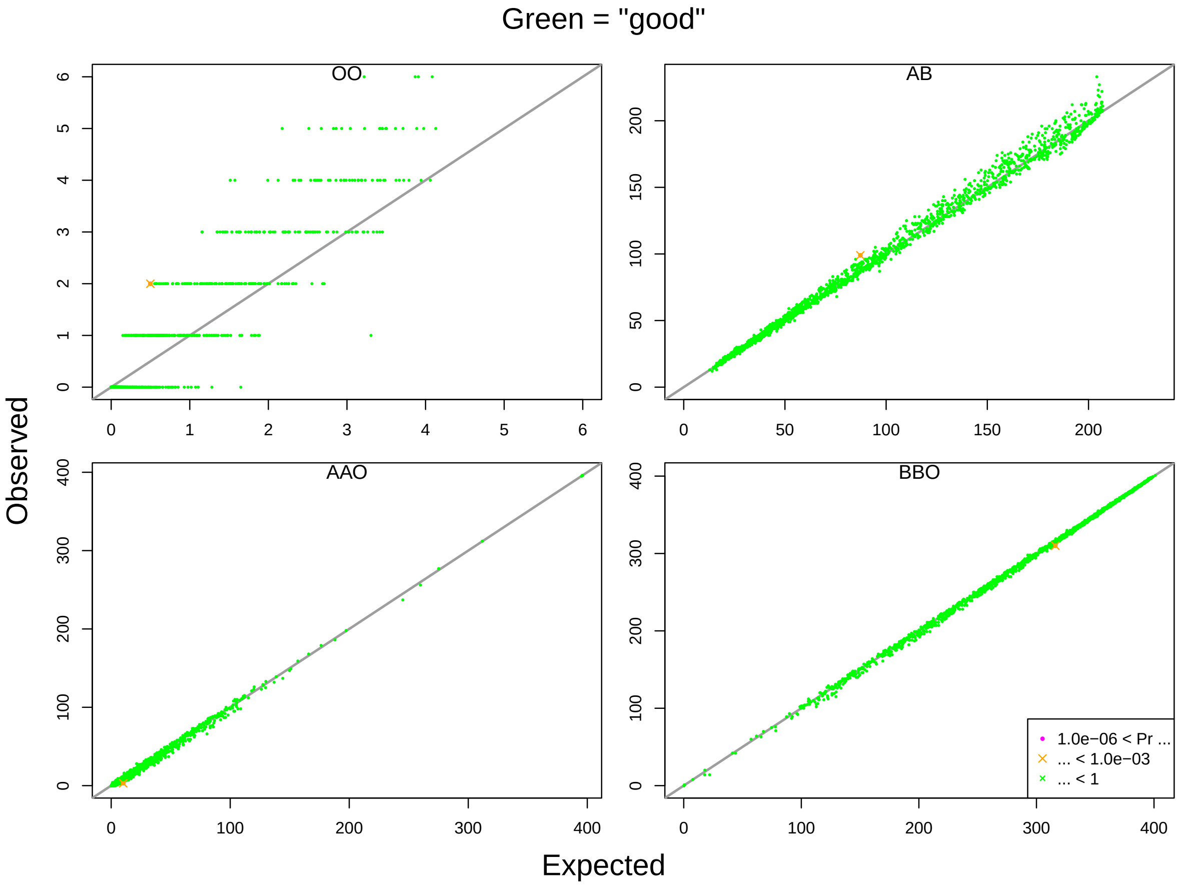

B

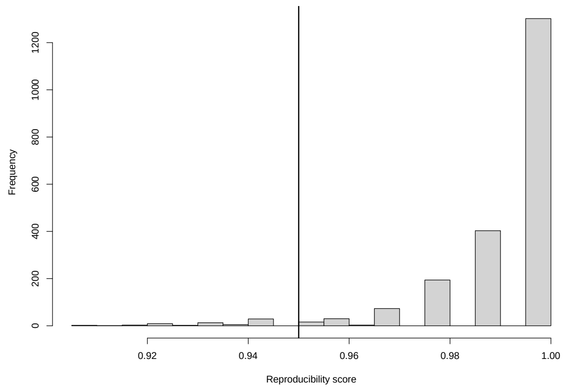

Figure S4 Quality control steps used for filtering for Hardy-Weinberg equilibrium and reproducibility. Panels in A) shows 4-way genotype counts against HWE expectations for 2,021 loci after filtering on allele frequency, null rates and HWE exact p-value at 1e-3. Points are coloured by the HWE exact test p-value with those less than 1e-6 already removed in these figures. Panel B) shows the distribution of reproducibility scores provided by DArT across 2,085 loci and the vertical line is the filtered value at 0.95.

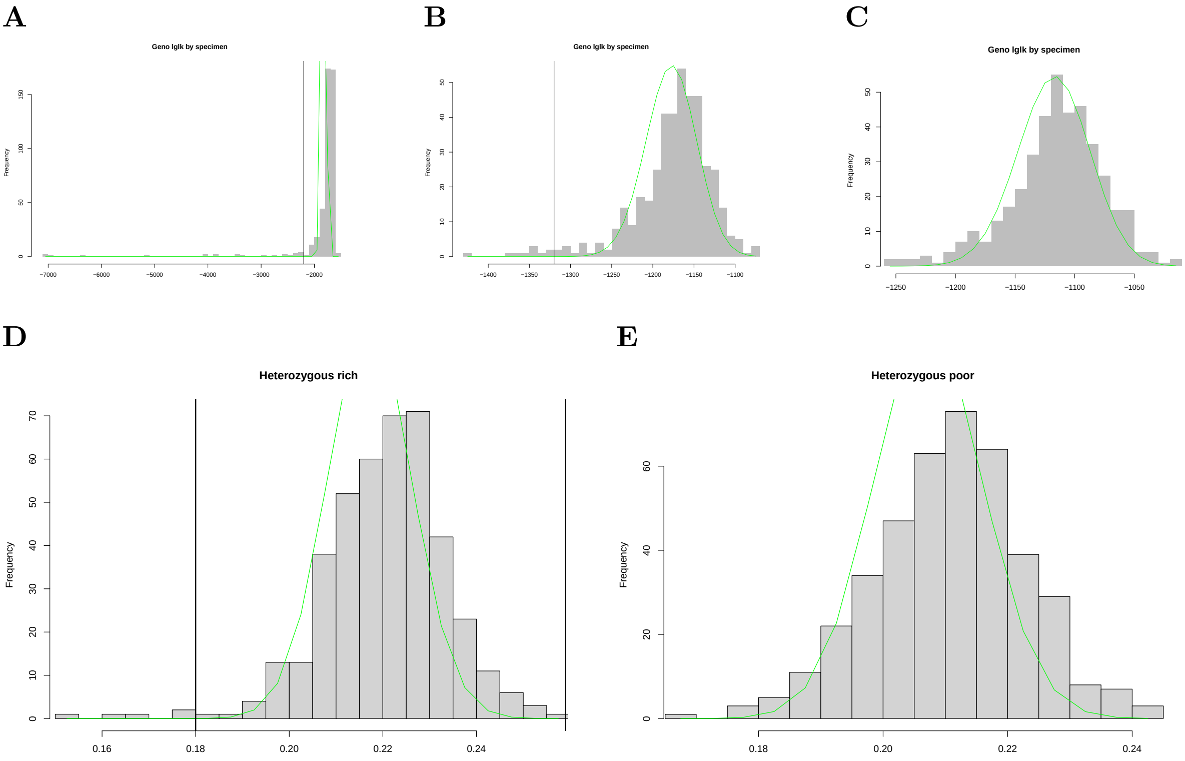

Figure S5 Quality control stepwise filtering for atypical genotypes and reproducibility. Panels A) to C) show the steps and filtering thresholds (vertical black lines) on the likelihood score distribution across individuals using the `ilglk_geno` function in the Kinference R package. Green solid lines represent the expected distribution given allele frequencies. The genotype likelihood procedure is done in steps with extreme outlier individuals removed and then allele frequencies and likelihood scores are computed again to interrogate the distribution. Panel C) shows the final step of the distribution. Fit for this filtering, step is typically not required to be of very high agreement as individuals are typically removed in down stream filtering steps. Panels D) and E) show the distributions of heterozygous poor and rich statistics across individuals generated using the `hetzminoo_fancy` function in Kinference. Panel D) shows the vertical line chosen for filtering on the rich statistic and no filter was used on the poor statistic. The green solid lines show the expected distribution for the statistic computed. The final distribution in panel E) contains 409 individuals, which formed the basis of the analysis in the main text.

B Half-sibling pair detection

The distribution of second-degree relatives contains two sources of unwanted relative pairs that have different methods for exclusion: (1) pairs of second-order relatives including grandparent-grandchild (GGP), and aunt-uncle/niece-nephew pairs; (2) contamination of the lower tail of the distribution with less related pairs. To address the first issue, we noted that the use of HSPs in the CKMR model requires knowledge of the birth year of both individuals. As only juveniles, sub-adults and adults of ages less than 36 months have accurate age information, only individuals in these age categories could be used. We further limited the maximum birth cohort gap between pairs to five years, which made the presence of GGPs now very unlikely because an absolute minimum birth-cohort gap of 58 months (2 x 24 months + 2 x 5 months gestation) would be required for a GGP to occur. Limiting the comparisons to only include individuals less than 36 months of age and with a threshold on the birth-cohort gap, showed a refined PLOD distribution (Figure 1).

To address the possible contamination of the lower tail of the pseudo-log-odds (PLOD) kinship statistic distribution with less related pairs, half-uncle/niece (referred to from here on as half-thiatic pairs (HTPs)) and half-cousins have coefficients of relatedness of 0.125 and 0.0625, respectively compared to 0.25 for HSPs. These kinship types have the potential to contaminate the lower tail of the HSPs PLOD distribution. Subsetting to juveniles and sub-adults does not exclude these kinship types because these individuals can be arbitrarily close in the birth-cohort gap. Therefore, to select a reliable set of HSPs we must decrease the likelihood of the chosen HSP set containing other kin pair types of lower relatedness, particularly HTPs. Following the principles set out in Bravington et al.[7] we: (1) picked a threshold that will exclude false positives such that we were very sure no HTPs are left in the HSP distribution; (2) defined HSPs to be pairs that came above the chosen threshold; (3) quantified the potential removal of some true HSPs as a result of setting the threshold to exclude HTPs and other less related pairs, and accounted for this in the model.

We implemented this strategy via the following steps: (1) we estimated the mean and

variance of the PLOD for unrelated individuals and validated these against the empirical distribution to check that the allele frequency prediction was valid; (2) estimated the mean PLOD for HSPs from allele frequencies and then estimated the variance of the HSP PLOD distribution by looking at those pairs above the expected HSP value; (3) estimated from allele frequencies the expected PLOD for HTPs using statistical genetics theory and assuming that the probability that two HTP individuals have exactly 0 or 1 alleles identity by descent is k0 = 0.75 and k1 = 0.25. We then estimated the variance of the HTPs relatives by using the predicted HSP variance to estimate the effective crossover rate and subsequent theory to estimate the variance for different expected genome sharing for different relative types. We further checked that the variance of the HTPs lies between the HSP and unrelated pair (UP) variance; (3) chose a cut-off PLOD value such that very few HTPs could lie above this PLOD value; (4) calculated the proportion of the HSP distribution that lies below the threshold and corrected the HSP kin probabilities by multiplying them by one minus this proportion.

Following this procedure, the theoretical mean and variance of the PLOD scores for the UPs were -45.5 and 85.7, respectively, which were very close to the empirical means and variances of -45.5 and 93.1, (Figure S1). The expected HSP PLOD score was 49.9 with an empirical variance of 155.9. The expected HTPs pairs PLOD score was 2.2 with an empirical variance of 132.7, which lies between the UP and HSP variance. If we assume HTPs are three times more common than HSPs Hillary et al.[6] then assuming we have 65 HSPs then we expect approximately 195 HTPs to be present. A PLOD score of 32 ensures that only 0.5% of HTPs (or < 1 HTP) could lie above this value given the assumption above that the mean of the HSP distribution is correct. At this PLOD threshold, 8.4% of the HSP distribution is excluded and thus in the CKMR model, the HSP probabilities were multiplied by 0.916 to correct for this discrepancy. At this threshold, 65 HSPs that have a cohort gap $\leqslant 5$ and have ages $\leqslant 36$ months were detected and summarised with their covariate data in Supplementary Table S2.

C Aunt/Uncle-Niece/Nephew kin pair rate simulation study

Implementation

To investigate the kinship patterns that could arise from hypothesised CIFF populations under varying assumptions, we wrote a forward simulation using the R programming language. The simulation is designed to mimic the CIFF population given the known biological constraints of the species. The goal was to understand the expected number of uncle/aunt - niece/nephew (referred to as full-thiatic pairs (FTPs)) relative to other kinship types in such a population.

The simulation generates a hypothetical CIFF population over generations with yearly time steps. The population is initialised with a set of founding individuals with the expected female-to-male sex ratio set at 50:50; the juvenile, sub-adult and adult proportions set at (0.58, 0.14, 0.28) to match the observed data proportions. The simulation begins in the year 1978 and evolves over 40 generations ending in the year 2018. In the base scenario, each year of the simulation contains one breeding event using all available adult pairs. A random subset dies each year with the total number of deaths taken as a proportion of the total population size, which is controlled by the mortality rate parameter δ. Individuals are allowed to age to an arbitrary value i.e., there is no fixed maximum age. The population is recorded each year for later sampling with the data structures sufficient to recover the kinship status between all individuals sampled.

We simulated the population under the following further assumptions that are based on the estimates obtained from the observed data. We set the initial population size to 4,500, which is the total population size that corresponds approximately to recent CMR study estimates of Todd[8]. The mortality rate parameter was tuned such that the population has a stable rate of change at a $\delta = 0.26$.

To investigate the expected kin patterns arising from an increased reproductive output from a subset of the male population we used the initial simulation structure but truncated the male breeding population size in each generation by $\pi \in (0, 1)$. For example, when π is set to 0.5 only 50% of the potential breeding males contribute to the next generation. We further allowed varying rates of maintenance of individuals in the domi-

nant male set. For example, if we use a 0.05 dominant male maintenance rate then only five per cent of the current dominant males are maintained for breeding in the subsequent generation. We fixed this dominant male maintenance rate to 0.8 in each simulation.

To study mate persistence we further allowed for mating pairs to be stored across generations and rotated at different rates. The mate persistence parameter controls the proportion of the prior generation's mating pairs that are randomly assigned new mates in the current generation. For example, of the pairs that bred last year and survived to this generation a value of 0.1 assigns 10% of the breeding pairs new partners from the available set of new adult super males.

We simulated populations across a grid of dominant male (0.2, 0.4, 0.6, 0.8) and mate persistence proportions (0.1, 0.3, 0.5, 0.7, 0.9). For each simulated population (20 combinations), the most recent five years were sampled (2014, 2015, 2016, 2017, 2018) under a mark-recapture sampling strategy (although lethal sampling can be performed) with 85 individuals sampled per year to arrive at a sample size of 425 individuals. Five years of sampling was chosen as that is the number of years available in the current study and the number of samples per year reflects the sample size taken in our study. For each of the scenarios, 10 simulation replicates were performed.

The results were summarised by visualising the observed number of MOPs, FOPs, MHSPs, PHSPs, FSPs, and FTPs. We plotted the observed number of FSPs and MHSPs to intersect the simulation results with the observed number of kin pairs.

Results

The simulation results suggest that FTPs track FSPs relatively closely except when mate persistence is very strong (Supp. Figure S6). The observed number of FSPs in the CIFF data set corresponds to approximate mate persistence proportions between 0.5 to 0.9. Similarly, the observed number of kin maps breeding male proportion corresponds to 0.6 to 0.8. In these ranges we expect the number of FTPs to be close to the number of FSPs. Therefore, overall for CIFFs, we expect FTPs to be a small proportion of the HSP pair set i.e., three on expectation.

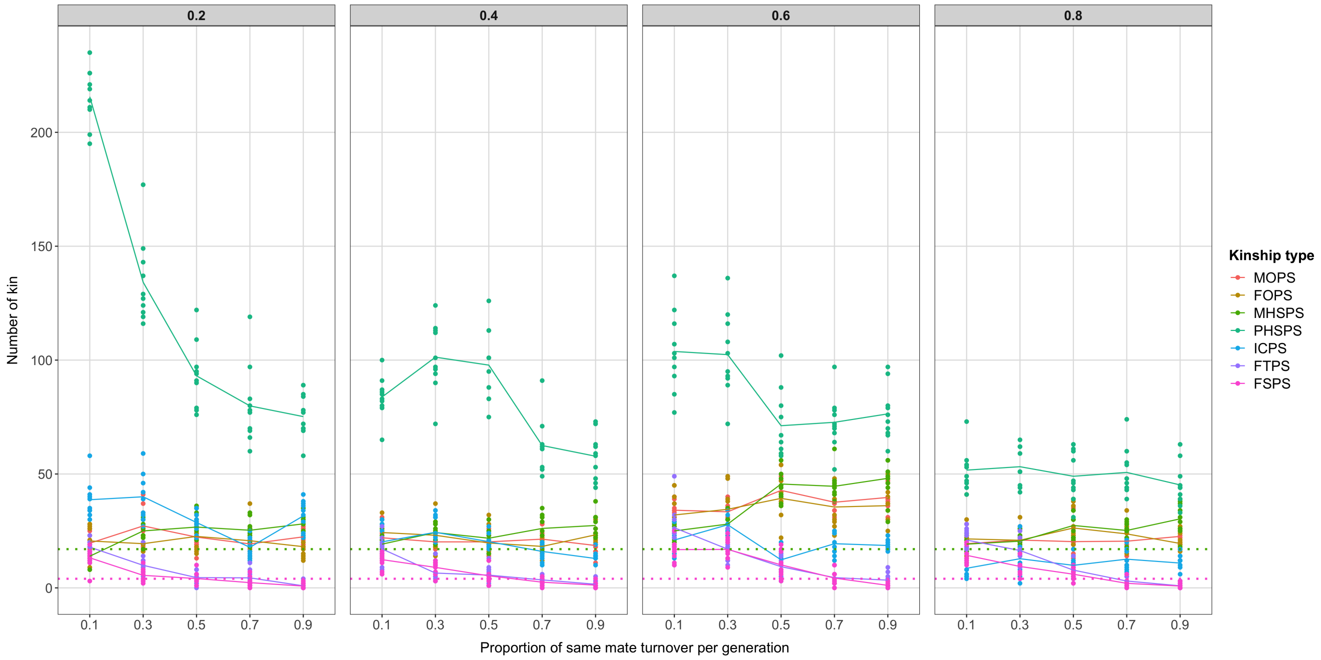

Figure S6 Summary of the number of kin of different types from forward simulation with a subset of more reproductively breeding males and mate persistence. The total sample size was 4,500 individuals. For each proportion of dominant males (panel labels) and mate persistence parameters (x-axis) 10 simulation replicates were performed. Each point is the number of kin pairs from each of the simulation scenarios. The number of intra-cohort pairs (ICPS) is reported which increases with the increased male reproductive output parameter. The dashed horizontal lines represent the number (3) of observed of FSPs (pink) and the number (17) of same-mtDNA haplotype HSPs (MHSPs) in the CIFF data set.

D Mitochondrial DNA sequencing and quality control

To investigate the sex of the shared parent for half-sibling pairs detected in the kinship analysis we used mitochondrial DNA (mtDNA) sequencing. This was performed for the set of individuals in the pairs that showed evidence to be first or second-degree relatives. First-degree relatives were included to assist in validation and discrimination between POPs and FSPs. To this end, leftover extracted DNA from the DArTseq procedure was used in the amplification of a fragment of the control region. It was expected that the CIFF has enough mtDNA diversity in this region based on prior studies[9]. Primers RodmtU 5'-GCTGAGGTTCTACTTAAACT-3' and RodmtL 5'-GAGATGTCTTATTTAAGGGG3'[10] were used to amplify a 461 base pair region spanning 452 bp of the beginning of the con-

trol region and 9 bp of the adjacent tRNA-Proline region. PCR was performed in 25 μL volumes containing final concentrations of 1 $\times$ MRT buffer (1 $\times$ Immolase buffer, 1.5 mM MgCl2, 0.8 mM dNTP mix, 0.05 mg/ml BSA), 0.24 M Betaine, 0.4% Dimethyl Sulphoxide, 0.4 μM of each primer and 2 U of IMMOLASE^{TM} DNA polymerase (Meridian Bioscience^{TM}). Thermocycling was performed as follows: initial denaturation at 94 °C for five minutes; 35 cycles of 94 °C for 30 seconds, 50 °C annealing for 30 seconds and 72 °C for two minutes; and a final extension of 72 °C for ten minutes. Amplicons were sequenced in both directions using the PCR primers by AGRF Ltd, Adelaide, South Australia. Sequences were edited and aligned manually in BioEdit version 7.2.5 software[11]. A set of 37 individuals was repeated to assess the error rate for the region of the mitochondrial genome sequenced. Quality control for sequencing errors for the start and end components of the reported sequences was performed and sequences were truncated across all individuals leaving 402 base-pair mtDNA sequences. A set of unique sequences (referred to as haplotypes) was determined across the set of sequences.

E Derivation of kin probabilities

Given the CIFF has been listed as critically endangered, tissue samples and measurements are taken and the individual is released. Furthermore, adult age is unknown. Therefore, we require a form for the parent-offspring probabilities that do not require adult age and encompass both the cases where the parent was sampled after the offspring (analogous to lethal sampling) and when the parent was sampled (potentially as a juvenile) before the offspring were sampled. The kinship probabilities are the building blocks of the pseudo-likelihood defined below and link the population dynamics model, and the associated parameters $\boldsymbol{\theta}$, with observed kinship for the pair of individuals. Typical base model parameters include adult abundance at time t, trend, and mortality/survival. For brevity in the following, we drop the $\boldsymbol{\theta}$ from the notation but they are implicit in the computations.

POP probabilities are split into mother-offspring pairs (MOPs) and father-offspring pairs (FOPs) for simplicity, as sex is known. Suppose we wish to find the probability that

individual i, sampled at time t_i, is the mother of juvenile j, born at time y_j. One way to derive these probabilities is via the notion of expected reproductive output (ERO)[7]. We assume once a female reaches maturity, she has a fixed fecundity that is independent of covariates such as age. We can then suppose, in general, the ERO of any mature female is β, and of $N_{♀}$ mature females is $\beta N_{♀}$, for some positive constant β. Then the probability that i is j's mother is equal to the expected *relative* reproductive output of i in the year of j's birth, y_j. This is given by the equation

$$\mathbb{P}(K_{ij} = \text{MOP}|\mathbf{z}_i, \mathbf{z}_j) = \frac{\mathbb{E}[R_i(y_j)|\mathbf{z}_i]}{\mathbb{E}[R_{♀}(y_j)]},$$

where $\mathbf{z}_i$ and $\mathbf{z}_j$ are the vector of observed covariates for individuals i and j, and includes t_i. $R_i(y_j)$ is the reproductive output of i at y_j and $R_{♀}(y_j)$ is the total reproductive output of all mature females at y_j. We define $v_.(.)$ and $m_.(.)$ to monitor the state of whether an individual was alive/vital and/or mature at a time point. For example, $v_i(t_i) = 1$ implies individual i was alive at t_i and $m_i(t_i) = 1$ implies individual i was mature at t_i. It is important to note that $\mathbb{E}[R_i(y_j)|\mathbf{z}_i] = \beta$ if i is both alive and mature at y_j. For CIFF we do not know adult age and thus y_i is unknown and not a component of $\mathbf{z}_i$. Implicit in the following derivation is that $v_i(t_i) = 1$ and $m_i(t_i) = 1$. Thus

$$\begin{aligned}\mathbb{E}(R_i(y_j)|t_i) &= \mathbb{E}\{R_i(y_j) \cdot \mathbb{I}[v_i(y_j) = 1 \cap m_i(y_j) = 1|t_i]\} \\ &= \beta \cdot \mathbb{P}[v_i(y_j) = 1 \cap m_i(y_j) = 1|t_i]\end{aligned}$$

This reduces the MOP probability to

$$\begin{aligned}\mathbb{P}(K_{ij} = \text{MOP}|\mathbf{z}_i, \mathbf{z}_j) &= \frac{\beta \cdot \mathbb{P}[v_i(y_j) = 1 \cap m_i(y_j) = 1|t_i]}{\beta \cdot N_{♀,y_j}} \\ &= \frac{\mathbb{P}[v_i(y_j) = 1 \cap m_i(y_j) = 1|t_i]}{N_{♀,y_j}}\end{aligned} \tag{1}$$

where $N_{♀,y_j}$ is the number of mature females at time y_j. Therefore, the MOP probability rests on establishing the numerator in terms of observables for the case when the mother is sampled after the juvenile and when the mother is sampled before.

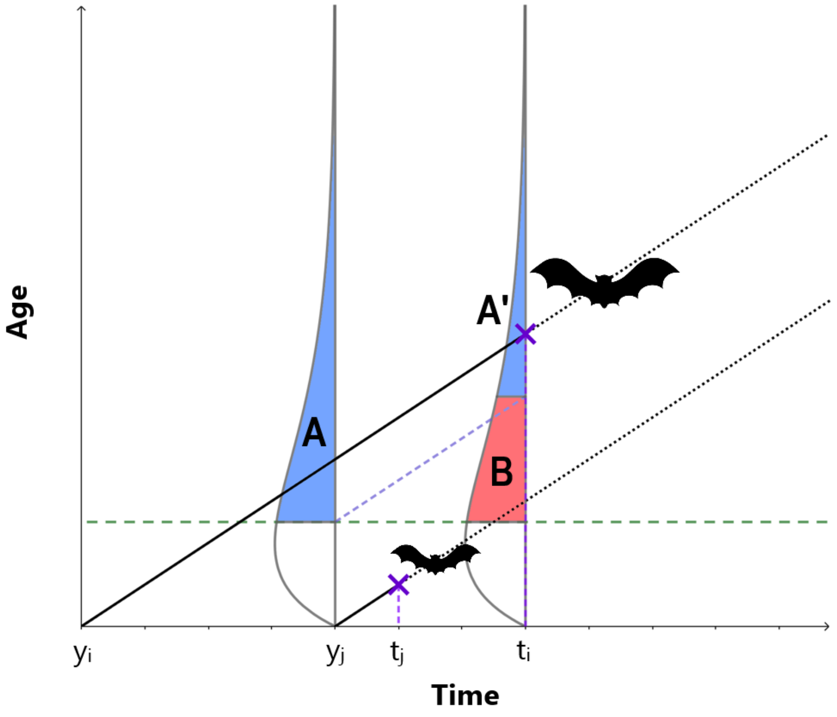

Figure S7 Lexigram illustrating a sampling situation where $y_j < t_i$. The green horizontal line indicates the age of maturity and a purple cross indicates sampling. A represents $N_{♀,y_j}$; A' represents $\phi(y_j, t_i)N_{♀,y_j}$; and $A' \cup B$ represents $N_{♀,t_i}$. For some individual i, sampled at t_i from $A' \cup B$, i is a member of A if i is a member of A'.

Case 1: $y_j < t_i$

If $y_j < t_i$, then the lethal and non-lethal sampling cases reduce to the same situation, which can be understood from the 'lexigram' in Figure S7. In this case, we use conditional probability to write

$$\mathbb{P}[v_i(y_j) = 1 \cap m_i(y_j) = 1|t_i] = \mathbb{P}\{[v_i(y_j) = 1|m_i(y_j) = 1]|t_i\}\mathbb{P}[m_i(y_j) = 1|t_i]$$

It is important to note that we are implicitly conditioning on $v_i(t_i) = 1$ and $m_i(t_i) = 1$. The logic is that of those mothers at t_i what's the probability that an individual was from the mature females at y_j. As any female is equally likely to be the mother of j at t_i then the probability that i was mature at y_j is the ratio of mature mothers from y_j that survived to t_i over the number of potential mothers at t_i (Figure S7 demonstrates this

pictorially). Therefore,

$$\mathbb{P}[m_i(y_j) = 1|t_i] = \frac{\phi(y_j, t_i) \cdot N_{♀,y_j}}{N_{♀,t_i}}$$

where ϕ is the survival function. Additionally, given i would have been mature at y_j and i is alive at t_i, she must have been alive at $y_j < t_i$. That is,

$$\mathbb{P}\{[v_i(y_j) = 1|m_i(y_j) = 1]|t_i\} = 1$$

Thus if $y_j < t_i$, the MOP probability is given by

$$\mathbb{P}(K_{ij} = \text{MOP}|\mathbf{z}_i, \mathbf{z}_j) = \frac{1 \cdot (\phi(y_j, t_i) \cdot N_{♀,y_j})/N_{♀,t_i}}{N_{♀,y_j}} = \frac{\phi(y_j, t_i)}{N_{♀,t_i}} \tag{2}$$

Case 2: $t_i < y_j$

In the non-lethal sampling for CIFF, i is released after sampling, so it is possible she is alive at y_j, where $t_i < y_j$ to give birth to j. In the previous MOP probability case, the uncertainty arose from the mother's maturity at the birth of the juvenile, which was backdated in time. In this case, $v_i(t_i) = 1$ and $m_i(y_j) = 1$ implies certainty of maturity at the birth of j given i survives from t_i to y_j. It is important to note that, due to the knowledge of juvenile age, we only consider individuals who could reach maturity by y_j as 'potential mothers', which guarantees $m_i(y_j) = 1$. In Figure S8 this is indicated by the exclusion of C from the sets i may have been sampled from. Therefore, the logic rests on a projection into the future with the uncertainty arising from survival of the mother. We consider the numerator of Equation 1

$$\mathbb{P}[v_i(y_j) = 1 \cap m_i(y_j) = 1|t_i] = \mathbb{P}\{[m_i(y_j) = 1|v_i(y_j) = 1]|t_i\}\mathbb{P}[v_i(y_j) = 1|t_i]$$

This means

$$\mathbb{P}[v_i(y_j) = 1|t_i] = \phi(t_i, y_j)$$

Furthermore,

$$\mathbb{P}\{[m_i(y_j) = 1|v_i(y_j) = 1]|t_i\} = 1$$

Thus, for non-lethal sampling when $t_i < y_j$, the MOP probability is given by

$$\mathbb{P}(K_{ij} = \text{MOP}|\mathbf{z}_i, \mathbf{z}_j) = \frac{\phi(t_i, y_j) \cdot 1}{N_{♀,y_j}} = \frac{\phi(t_i, y_j)}{N_{♀,y_j}} \tag{3}$$

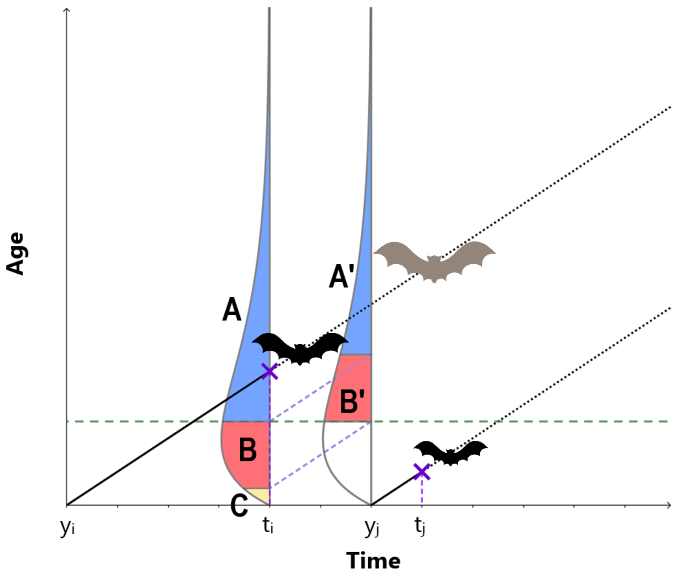

Figure S8 Lexigram illustrating a non-lethal sampling situation where $t_i < y_j$. The green horizontal line indicates the age of maturity and a purple cross indicates sampling. A represents $N_{♀,t_i}$; A' represents $\phi(t_i, y_j)N_{♀,t_i}$; $A' \cup B'$ represents $N_{♀,y_j}$; and $A \cup B$ represents $N_{♀,y_j}/\phi(t_i, y_j)$. For some individual i, sampled at t_i from $A \cup B$, i is a member of $A' \cup B'$ if and only if i survives from t_i to y_j. Note individuals in C are alive at t_i, but will not mature by y_j.

Case 3: half-sibling pairs

The statement of the calculation is presented in Bravington et al.[7] but we include the details here for completeness. Similar logic can also be applied to derive the HSP probabilities. Pairs of juveniles are considered for maternal half-sibling pairs (MHSPs) and paternal half-sibling pairs (PHSPs) separately. Suppose we wish to find the probability that i and j, born in y_i and y_j respectively, with $y_i < y_j$, form an MHSP. We can take some unknown but fixed individual m as the true mother of i. Thus we know m was alive and mature at y_i, which is analogous to m having been sampled at y_i. This means the probability that i and j form an MHSP is simply the probability that m is the mother of j, with $t_m = y_i < y_j$. Thus by adapting the MOP probability from Equation 3, the MHSP probability is given by

$$\mathbb{P}(K_{ij} = \text{MHSP}|\mathbf{z}_i, \mathbf{z}_j) = \frac{\phi(y_i, y_j)}{N_{\female, y_j}} \tag{4}$$

The FOP and PHSP probabilities are easily adapted from the MOP and MHSP probabilities respectively. See Bravington et al.[7] for a further detailed derivation.

F Derivation of sibling probability and Binomial class log pseudo-likelihood

The presence of full sibling pairs and intra-cohort half-sibling pairs implies that there is evidence for site and mate fidelity along with male reproductive skew in the CIFF population. Therefore, to integrate the presence of full siblings and to differentiate maternal and paternal half-sibling pairs, we derive the probability of being a sibling pair for any comparison between individuals i and j. When FSPs are rare it may be reasonable to treat the events of sharing a mother and a father as independent events, however, for CIFF this is unlikely. Therefore, the potential dependence needs to be incorporated appropriately. Furthermore, FSPs are not required to be controlled for the false-negative rate from thresholding the kinship statistics (PLOD) because their kinship statistics do not overlap with lower-order kin when sufficient markers are available.

Therefore, for sibling pairs (SPs) the $\mathbb{P}(K_{ij} = \text{SP}|\mathbf{z}_i, \mathbf{z}_j; \boldsymbol{\theta})$ needs to cover the three cases (FSP, MHSP, PHSP) and incorporate the notion of passing the PLOD threshold to be considered a sibling pair. For brevity, we again drop the $\boldsymbol{\theta}$ in the following notation. This broad-scale SP probability is then corrected for the uncertainty in sharing a haplotype, which assigns a weighted HSP probability to sharing a mother or a father.

We define the following variables and events:

- Let M be the event that a pair shares a mother and $\bar{M}$ be the event that the mother is not shared.
- Let P be the event that a pair shares a father and $\bar{P}$ be the event that the father is not shared.
- Let D be the event that we are sure a pair are *genetically* siblings i.e., has the correct covariate information and kinship statistic is greater than the threshold value for HSPs. For example, in this work, this corresponds to the PLOD threshold at 34.
- Let ν be the false-negative rate for HSPs, which is estimated from the lower tail of the thresholded distribution estimated from the HSP component of the PLOD

score distribution (see Figure 1 main text).

- Let h_i be the observed mtDNA haplotype for ith individual.
- Let $f(h_i)$ be the population frequency of the haplotype for individual i, estimated from the distribution of haplotypes in the population.
- Let α represent the mate fidelity parameter that increases the probability of sharing a mother and a father incorporating mate persistence relative to individuals choosing new mates.

Using the above statements the probability $\mathbb{P}(K_{ij} = \text{SP}|\mathbf{z}_i, \mathbf{z}_j)$ is equivalent to (implicit that the events M, P and D are over the pair of individuals i and j)

$$\begin{aligned}
\mathbb{P}(M \cup P, D|\mathbf{z}_i, \mathbf{z}_j) &= \mathbb{P}(M \cap P, D|\mathbf{z}_i, \mathbf{z}_j) + \mathbb{P}(M \cap \bar{P}, D|\mathbf{z}_i, \mathbf{z}_j) + \mathbb{P}(\bar{M} \cap P, D|\mathbf{z}_i, \mathbf{z}_j) \\
&= \mathbb{P}(M \cap P|\mathbf{z}_i, \mathbf{z}_j)\mathbb{P}(D|M \cap P, \mathbf{z}_i, \mathbf{z}_j) + \\
&\quad \mathbb{P}(M \cap \bar{P}|\mathbf{z}_i, \mathbf{z}_j)\mathbb{P}(D|M \cap \bar{P}, \mathbf{z}_i, \mathbf{z}_j) + \\
&\quad \mathbb{P}(\bar{M} \cap P|\mathbf{z}_i, \mathbf{z}_j)\mathbb{P}(D|\bar{M} \cap P, \mathbf{z}_i, \mathbf{z}_j) \\
&= \mathbb{P}(M \cap P|\mathbf{z}_i, \mathbf{z}_j) \times 1 + \\
&\quad \mathbb{P}(M \cap \bar{P}|\mathbf{z}_i, \mathbf{z}_j) \times (1 - \nu) + \\
&\quad \mathbb{P}(\bar{M} \cap P|\mathbf{z}_i, \mathbf{z}_j) \times (1 - \nu) \quad , \qquad (5)
\end{aligned}$$

where the multiplier for the FSP probability is 1 because the position of FSPs in the PLOD statistic distribution is not contaminated from lower degree relatives i.e., all FSPs are certainly above the PLOD sibling threshold.

To compute the first component of Equation (5) we incorporate the potential for mate fidelity across breeding seasons using the model

$$\mathbb{P}(M \cap P|\mathbf{z}_i, \mathbf{z}_j) = \mathbb{P}(P|\mathbf{z}_i, \mathbf{z}_j) \times \text{logit}^{-1}\{\alpha + \text{logit}[\mathbb{P}(M|\mathbf{z}_i, \mathbf{z}_j)]\} \quad , \qquad (6)$$

where α models mate persistence and $\mathbb{P}(P|\mathbf{z}_i, \mathbf{z}_j)$ and $\mathbb{P}(M|\mathbf{z}_i, \mathbf{z}_j)$ are computed from Equation (4) i.e., are the unadjusted maternal and paternal half-sibling. For this to

occur the sibling pair's father and mother have to survive and have a non-zero breeding probability. In particular, if pair i and j are from the same birth cohort then $\mathbb{P}(M|\mathbf{z}_i, \mathbf{z}_j) = 0$ and Equation (6) reduces to zero, which is required given females can have at most one pup per year (excluding rare twin events).

To compute the second and third components of Equation (5) we require Equation 6 and have

$$\mathbb{P}(M \cap \bar{P}|\mathbf{z}_i, \mathbf{z}_j) = \mathbb{P}(M|\mathbf{z}_i, \mathbf{z}_j) - \mathbb{P}(M \cap P|\mathbf{z}_i, \mathbf{z}_j) \text{ and} \tag{7}$$

$$\mathbb{P}(\bar{M} \cap P|\mathbf{z}_i, \mathbf{z}_j) = \mathbb{P}(P|\mathbf{z}_i, \mathbf{z}_j) - \mathbb{P}(M \cap P|\mathbf{z}_i, \mathbf{z}_j) \ . \tag{8}$$

Therefore, the computation of the sibling probability (Equation (5)) can be made using the combination of Equations (4), (6), (7) and (8).

For each sibling pair, we have further information about the attribution of information to the sex component of the demographic model stored in the mtDNA haplotype for each individual. Furthermore, a pair sharing haplotypes does not imply that the individuals share a mother. If high-frequency mtDNA haplotypes are present in the population a HSP may share a father but by chance share an mtDNA haplotype because each of their distinct mothers by chance had the high-frequency haplotype.

To incorporate these notions, we condition each sibling pair on the mtDNA haplotype of individual i and implicitly on the fact that the pair are definitely siblings. For each pair we have three mutually exclusive outcomes i.e., pair is an FSP ($M \cap P$), MHSP ($M \cap \bar{P}$) or PHSP ($\bar{M} \cap P$).

To incorporate the uncertainty in haplotype sharing and maternal/paternal status, we consider H_j, the mtDNA haplotype of the second individual, as a random variable. If the pair is an FSP then $H_j = h_i$, which is checked as part of quality control and attribution of FSP status, and thus H_j is uninformative in this case.

These equations condition on definitely being a sibling i.e., on the joint event $\{M \cup$

$P, D\}$ summarised by Equation (5). For FSP we have that

$$\mathbb{P}(M \cap P|\mathbf{z}_i, \mathbf{z}_j, h_i, \{M \cup P, D\}) = \frac{\mathbb{P}(M \cap P|\mathbf{z}_i, \mathbf{z}_j)}{\mathbb{P}(M \cup P, D|\mathbf{z}_i, \mathbf{z}_j)} \quad . \tag{9}$$

For HSPs, the calculation incorporates the mtDNA information and is

$$\mathbb{P}[(M \cap \bar{P}) \cup (\bar{M} \cap P), H_j|\mathbf{z}_i, \mathbf{z}_j, h_i, \{M \cup P, D\}] = \tag{10}$$

$$\mathbb{P}[(\bar{M} \cap P)|\mathbf{z}_i, \mathbf{z}_j, \{M \cup P, D\}] \times \mathbb{P}[H_j|(\bar{M} \cap P), h_i] \; +$$

$$\mathbb{P}[(M \cap \bar{P})|\mathbf{z}_i, \mathbf{z}_j, \{M \cup P, D\}] \times \mathbb{P}[H_j|(M \cap \bar{P}), h_i] =$$

$$\frac{\mathbb{P}(\bar{M} \cap P|\mathbf{z}_i, \mathbf{z}_j)}{\mathbb{P}(M \cup P, D|\mathbf{z}_i, \mathbf{z}_j)} \times f(h_j) + \begin{cases} \frac{\mathbb{P}(M \cap \bar{P}|\mathbf{z}_i, \mathbf{z}_j)}{\mathbb{P}(M \cup P, D|\mathbf{z}_i, \mathbf{z}_j)} & H_j = h_i \\ 0 & H_j \neq h_i \end{cases} \quad . \tag{11}$$

Given these considerations and to improve computation efficiency, we implemented the following covariate-class Binomial penalised log pseudo-likelihood

$$\ell_P(\boldsymbol{\theta}) \propto \sum_y \sum_t \sum_s \{k_{yts} \log[\mathbb{P}(\mathrm{POP})_{yts}] + [c_{yts} - k_{yts}] \log[1 - \mathbb{P}(\mathrm{POP})_{yts}]\} \; +$$

$$\sum_{y_1} \sum_{y_2} \{k_{y_1 y_2} \log[\mathbb{P}(\mathrm{SP})_{y_1 y_2}] + [c_{y_1 y_2} - k_{y_1 y_2}] \log[1 - \mathbb{P}(\mathrm{SP})_{y_1 y_2}]\} \; +$$

$$\sum_{n_{\mathrm{FSP}}} \log[\mathbb{P}(M \cap P|\mathbf{z}_i, \mathbf{z}_j, h_i, \{M \cup P, D\})] \; +$$

$$\sum_{n_{\mathrm{HSP}}} \log[\mathbb{P}[(M \cap \bar{P}) \cup (\bar{M} \cap P), H_j|\mathbf{z}_i, \mathbf{z}_j, h_i, \{M \cup P, D\}]] \; -$$

$$\frac{(\delta_{♀} - \mu_{\delta_{♀}})^2}{2\sigma^2_{\delta_{♀}}} \quad , \tag{12}$$

where y iterates over all birth cohorts present in the data, t iterates over all sampling years present in the data, s iterates over sex, c_{yts} represents the number of comparisons in the covariate class such that $\mathbf{z}_i = (t, s)$ and $\mathbf{z}_j = y$ i.e., the sampling year of the older individual is y and the sex is female for MOP and male for FOP. The coefficient k_{yts} represents the number of detected kin of a particular type (MOP, FOP) with a sampling year t for the potential adult and birth cohort y for the potential juvenile and similarly

for $k_{(y_1,y_2)}$ the number of siblings detected with older individual born in year y_1 and younger individual born in year y_2. The POP and SP kinship probabilities (written with compressed notation) for components one and two of Equation (12) are computed as per Equations (2), (3), and (5). Lines three and four of Equation (12) incorporate the extra information implicitly conditioned on in the broad sibling pair probability calculation, which includes mtDNA haplotype frequency information that is informative about sex-specific parameters (see Supplementary Equations (9) and (11)) and the prior probability of mtDNA haplotypes through observed frequencies in the data. The final line includes the necessary elements of the logged Gaussian prior for the mortality parameter, which is included or dropped over the classes of models fitted.

G Reduced female-only model

We would like to fit a female-only population dynamics model to just the maternal kin-pairs (MOPs and maternal SPs i.e., a sib-pair that shares a mother, regardless of the father) because it has fewer parameters and we are more comfortable with the female-related biological assumptions. The process serves as a common-sense check on results for the more elaborate models that attempt to tackle all the phenomena properly, and it is often helpful to see explanations and results from a simpler model first.

Since we cannot cleanly separate the maternal-descent kin from the rest, we will have to do it in a less complete yet principled way. We need to use empirically averaged information to adjust the numbers of maternally-linked sibling pairs (POPs are simple as we only use comparisons against female adults).

There are two issues that this process requires

1. We cannot cleanly split MHSP from PHSP using mtDNA, because a PHSP could have shared haplotypes just by chance. For CIFF, we observed one haplotype with a high frequency in the individuals analysed ($\approx 20\%$)/ Therefore, in this instance, the probability of two individuals sharing this haplotype by chance in the population is $\approx 0.21^2 = 0.04$.

2. FSPs as well as MHSPs share a mother, and have similar "maternal dynamics". However, FSPs are fully detectable, whereas MHSPs have only approximately a 92% chance of exceeding the kinship PLOD statistics threshold.

Dealing with these two issues in order. Firstly, we address the discrepancy arising from uncertain mtDNA haplotype sharing. We let z be the covariates of the pair, and θ the demographic parameters. We know that

$$\mathbb{P}\left[\text{same-mtDNA HSP}|\mathbf{z}_i,\mathbf{z}_j;\boldsymbol{\theta}\right] = \mathbb{P}\left[\text{MHSP}|\mathbf{z}_i,\mathbf{z}_j\right] + \mathbb{P}\left[\text{PHSP}|\mathbf{z}_i,\mathbf{z}_j\right] \times \sum_{h\in\text{haplos}} f_h^2 \tag{13}$$

$$\mathbb{P}\left[\text{diff-mtDNA HSP}|\mathbf{z}_i,\mathbf{z}_j\right] = \mathbb{P}\left[\text{PHSP}|\mathbf{z}_i,\mathbf{z}_j\right] \times \left(1 - \sum f_h^2\right), \tag{14}$$

where f_h is the frequency of mtDNA haplotype h. Note the dropping of $\boldsymbol{\theta}$ in the notation

after Equation (13).

Now, we don't know $\mathbb{P}[\text{MHSP}|...]$ nor for PHSP, but *empirically* we do know the ratio of same-to-diff mtDNA HSPs (N.B. that, importantly, MHSPs and PHSPs have the *same* probability of exceeding the PLOD threshold). If we assume the pattern of same-to-diff with respect to covariates (e.g., birth dates) is the same then we can assume

$$\mathbb{P}[\text{PHSP}|\mathbf{z}_i, \mathbf{z}_j] \approx \tau \mathbb{P}[\text{MHSP}|\mathbf{z}_i, \mathbf{z}_j] \tag{15}$$

for some unknown τ. Then (13) and (14) become

$$\mathbb{P}[\text{same-mtDNA HSP}|\mathbf{z}_i, \mathbf{z}_j] = \mathbb{P}[\text{MHSP}|\mathbf{z}_i, \mathbf{z}_j]\,(1 + \tau \sum f_h^2) \tag{16}$$

$$\mathbb{P}[\text{diff-mtDNA HSP}|\mathbf{z}_i, \mathbf{z}_j] = \mathbb{P}[\text{MHSP}|z, \theta]\,\tau \left(1 - \sum f_h^2\right) \tag{17}$$

$$\implies \frac{\mathbb{E}[\#\textbf{detected}\text{ same...}]}{\mathbb{E}[\#\textbf{detected}\text{ diff...}]} = \frac{1 + \tau \sum f_h^2}{\tau\,(1 - \sum f_h^2)}. \tag{18}$$

For the last line, we use the fact that detection probability (i.e., of crossing PLOD threshold) is the same for both types of HSP, as is the number of comparisons. Then we plug the two observed numbers-of-detected-HSPs into (18), along with $\sum f_h^2$, which we know, to get an estimate of $\widehat{\tau}$. Then, in the model-fitting code, we can substitute $\widehat{\tau}$ and $\sum f_h^2$ into (16) and (17) so that thereafter we only need a formula for the demographic $\mathbb{P}[\text{MHSP}|\mathbf{z}_i, \mathbf{z}_j]$ (not its PHSP counterpart) in order to compute the same-mtDNA and diff-mtDNA probabilities.

Broadly, this process second-guesses the approximate effect, on the part of the model and the data that we are interested in, of fitting a correct full model to all the data. If we somehow did fit the full model, it would come up with an equivalent τ, which may depend on z, rather than being constant across z as we assume; e.g. PHSP $\div$ MHSP might depend on the birth gap) which would have to basically reproduce the observed same-to-diff ratio. The full model would likely do so more efficiently, but we expect it to be fundamentally the same. We do not propagate uncertainty associated with $\widehat{\tau}$ but we expect the influence on the inference to be small. Because this process is a check rather than formal inference we did not pursue this further.

Secondly, we address the issue arising from the detectability of FSPs versus MHSPs. From a female-adult-demographic perspective, FSPs and MHSPs are indistinguishable but the detection probability differs. FSPs are certain to be detected, but HSPs have only a (known) probability γ of exceeding the PLOD threshold ($\gamma \approx 0.92$ in this study). Then

$$\mathbb{P}\left[i, j \text{ are } \textbf{detected} \text{ as having same Mother} | \mathbf{z}_i, \mathbf{z}_j\right] = \gamma \times \mathbb{P}\left[K_{ij} = \text{MHSP} | \mathbf{z}_i, \mathbf{z}_j\right] + 1 \times \mathbb{P}\left[K_{ij} = \text{FSP} | \mathbf{z}_i, \mathbf{z}_j\right] \quad (19)$$

Again, the basic idea is that we compute an overall empirical ratio

$$\widehat{\xi} = \frac{\#\text{FSP}}{\#\text{MHSP}}$$

and plug that into (19), using the approximation that the ratio FSP:MHSP is independent of z, which won't be strictly true; mate swapping and mortality apply. That implies

$$\mathbb{P}\left[ij \text{ detected as Maternal sibs} | \mathbf{z}_i, \mathbf{z}_j\right] = \mathbb{P}\left[K_{ij} = \text{MHSP} | \mathbf{z}_i, \mathbf{z}_j\right] \left(\gamma + \widehat{\xi}\right) \quad (20)$$

The minor complications are that we don't detect all HSPs; we just detect same/diff-mtDNA, not maternal/paternal sibling pairs. However, we know from (16) that, since detection-probability is the same for all half-siblings (and since the number of comparisons is the same):

$$\mathbb{E}\left[\#\text{detected MHSP}\right] = \mathbb{E}\left[\#\text{detected same-mtDNA-HSP}\right] / \left(1 + \widehat{\tau} \sum_h f_h^2\right) \quad (21)$$

so that we end up with

$$\begin{aligned}\widehat{\xi} &= \frac{\#\text{FSP}}{\#\text{MHSP}} \approx \frac{\#\text{FSP}}{(\#\text{detected-MHSP}/\gamma)} \\ &= \gamma \frac{\#\text{FSP}}{(\#\text{detected-same-mt-HSP}/\left(1+\widehat{\tau}\sum_h f_h^2\right))} \\ &= \gamma \left(1+\widehat{\tau}\sum_h f_h^2\right) \frac{\#\text{FSP}}{\#\text{detected-same-mt-HSP}} \end{aligned} \tag{22}$$

Then we can plug $\widehat{\xi}$ into (20), so that we again only need the formula for demographic $\mathbb{P}[\text{MHSP}|z,\theta]$, and can compress the relevant kinship outcomes to (FSP or detected-same-mtDNA).

In summary, we estimate τ, by rearranging (18) and plugging in the observations. We estimate $\sum_h f_h^2$ by taking the unique haplotypes in the population and summing their observed squared frequency. For ξ, we use (22) and plug in $\widehat{\tau}$ and $\sum_h f_h^2$ and the number of FSPs and same mt-DNA half-sibling pairs.

We then replace (5) with (20) and only include in the mtDNA correction the FSPs and the same mtDNA half-sibling pairs. We keep the prior on the mortality parameter as described in the main text in likelihood equation (12) and remove the male component of the parent-offspring pair likelihood. Estimates for $\boldsymbol{\theta} = (N_{♀,t_0}, \varrho_{♀}, \delta_{♀})'$ are arrived at via maximising the pseudo-likelihood using `nlminb` function in the R programming language with the gradient supplied to the optimisation routine using algorithmic differentiation via the Template Model Builder.

Results

We initially estimated the three primary entities τ, $\sum_h f_h^2$ and ξ. For τ we have

$$\widehat{\tau} = \frac{1}{\frac{\mathbb{E}[\#\textbf{detected}\text{ same...}]}{\mathbb{E}[\#\textbf{detected}\text{ diff...}]}(1-\sum f_h^2) - \sum f_h^2}$$

where $\sum f_h^2 \approx 0.056$. Plugging in

$$\widehat{\tau} = \frac{1}{\frac{17}{26}(1 - 0.056) - 0.056} \approx 1.78$$

For ξ we have

$$\widehat{\xi} = \gamma \left(1 + \widehat{\tau} \sum_h f_h^2\right) \frac{\#\text{FSP}}{\#\text{detected-same-mt-HSP}}$$
$$= 0.916 * (1 + 1.78 * 0.056) * (3/17) \approx 0.178$$

These quantities were then used to estimate adult female abundance, mortality and trend. The highest precision year was 2017 with an estimate of the total female abundance of 1,948 (95% CI - (965, 3,933)) (Table S5). Estimates of mortality were similar to those in the broad (male and female) model but the trend point estimate was more negative. The uncertainty in both these estimates was very large with Figure S9 showing some resampled expected trends given this level of uncertainty. Model fit was adequate but showed borderline p-values at a 5% level when computing the probability that the observed values arose from a Poisson distribution with the expected number of kin computed under the estimated model (Tabel S6).

Overall, the results from the reduced model appear consistent with those observed in the more complex model that includes male dynamics.

Parameter	Estimate	SE	95% CI lower	95% CI upper
$N_{♀,t_0}$	3,250	2,699	639	16,544
$N_{♀,t_{2016}}$	2,214	828	1,064	4,608
$N_{♀,t_{2017}}$	1,948	698	965	3,933
$\delta_{♀}$	0.232	0.159	0.061	0.89
$\varrho_{♀}$	-0.102	0.207	-0.53	0.28

Table S5 Summary of reduced female-focussed CKMR model parameter estimates and uncertainty. Hessian-based standard errors (SE) are reported from TMB with 95% confidence intervals using $z_{1-0.05/2}$ as the SE multiplier constructed and then transformed for those parameters estimated on the log or logit scale.

Cohort gap	0	1	2	3	4	5	6	Total
Parent-offspring								
Maternal	–	0/1.53	0/1.47	1/0.926	0/ 0.596	0/0.088	0/0.076	1/4.68
Siblings								
Full	–	1/1.40	2/0.822	0/0.323	0/0.084	0/0.025	–	3/2.65
Maternal half	–	6/6.47	6/3.80	3/1.49	2/0.389	0/0.114	–	17/12.3

Table S6 Summary of observed versus expected kin. Cohort gap corresponds to the difference between the birth year of the juvenile and the capture year of the adult for POPs and the two birth cohort years for SPs. Each cell shows the number of observed numbers versus (/) the expected number of kin, computed as the number of combinations × kinship probability computed under the maximum likelihood estimates of $\boldsymbol{\theta}$. The sibling row was differentiated by sex for half-sibling pairs by taking those individuals that share an mtDNA haplotype as maternal.

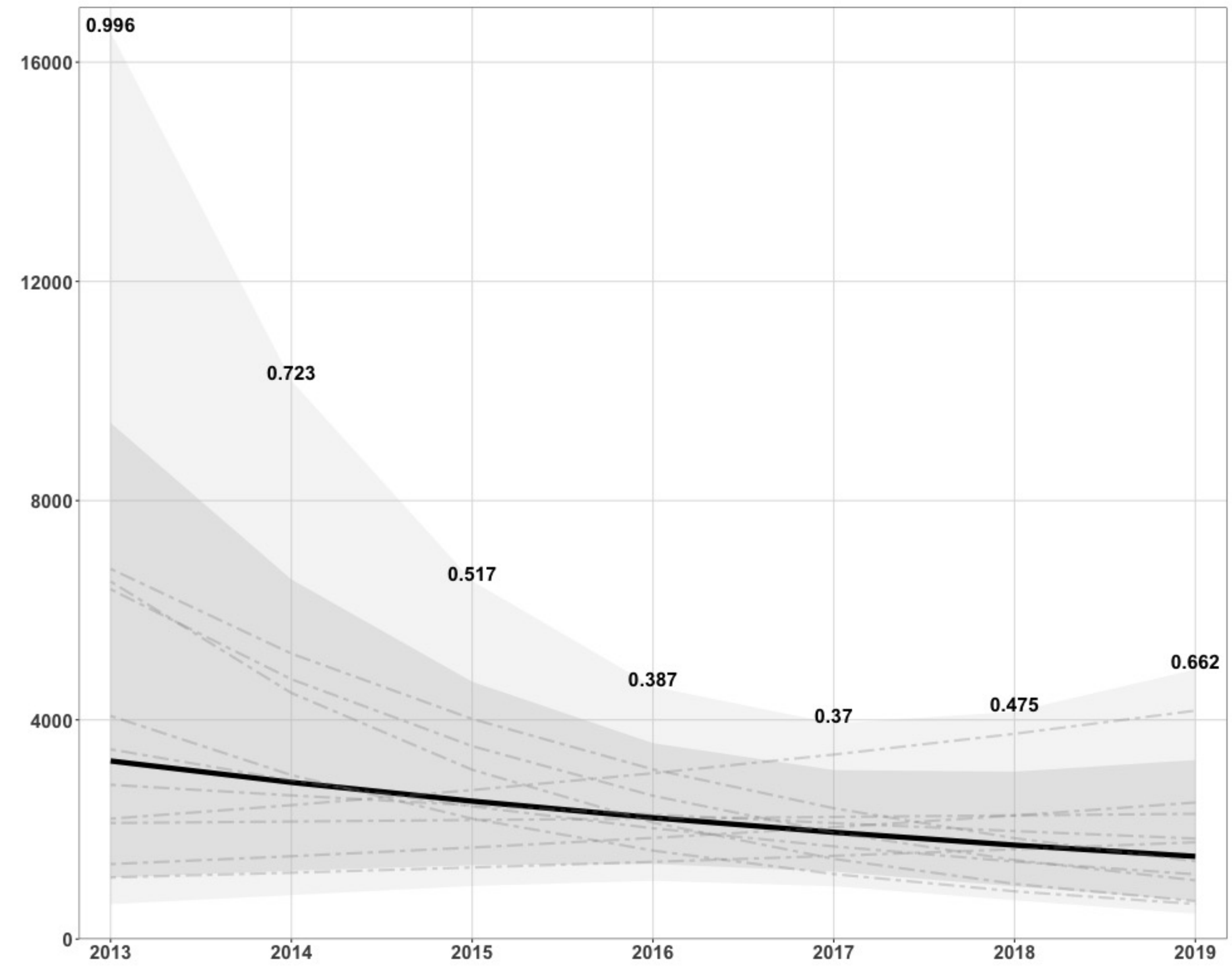

Figure S9 Estimated trend in total female abundance from reduced female-only model. Time series of total female abundance estimated by CKMR method shown as a dark solid line. Confidence intervals are depicted with grey shaded areas with 95% external and 80% internal. The coefficient of variation for each year's abundance estimate is reported as bold values above each upper interval estimate. Ten sampling importance resampled *worms* are added to show variability in trends generated from parameter estimates.

H Sensitivity of results over alternate models

We present results for the four cases that all have an adequate model fit and are biologically plausible. The following two-by-two table depicts the cases over the parameters that vary over the models and the order of presentation in the subsequent summaries,

	$\delta_{♀,♂}$ prior	
$\delta_{♂*}$	$0, 0$	$0, 1$.
	$1, 0$	$1, 1$

The cases where the mortality $\delta_{♀,♂}$ prior are 1 implies that a prior for adult female and male mortality was used, which had the expectation at the adult female value taken from Todd[8]. The values used after transformation were (μ=0.443, SD=0.206) corresponding to the transformed weighted (recapture sample size per year) averaged of the adult female survival values of (μ=0.643, SD=0.134) reported in Todd[8]. The adult female value was used because there was uncertainty in the contribution from male reproductive skew in the prior male estimates.

The cases where mortality for the breeding males $\delta_{♂*} = 1$ correspond to when we disconnect the mortality for the set of reproductive males. In the model, this corresponds to computing the paternal half-sibling probability using a mortality parameter just for this kinship type. This investigates whether reproductively successful males differ in their mortality from the total set of males and females.

The parameter π models the proportion of the total adult male set that is reproductively successful. That is, the number of males on the denominator in the paternal HSP calculation is $\pi \times N_{♂,t_0}$. The parameter $\varrho_{♀,♂}$ is the common population rate of increase parameter and α models mate persistence.

A

GoF = 4.91

Year gap	1	2	3	4	5	6	Total
Parent-offspring							
Maternal	0/1.47	0/1.36	1/0.86	0/0.56	0/0.08	0/0.07	1/4.41
Paternal	3/2.16	2/1.45	0/0.78	0/0.48	0/0.07	0/0.06	5/5
Siblings							
Full	1/1.55	2/0.77	0/0.26	0/0.06	0/0.01	-/-	3/2.64
Maternal half	6/6.53	6/4	3/1.61	2/0.44	0/0.13	-/-	17/12.71
Paternal half	15/17.2	10/10.3	2/4.08	0/1.11	1/0.32	-/-	28/33.06

B

GoF = 4.92

1	2	3	4	5	6	Total
0/1.6	0/1.39	1/0.81	0/0.48	0/0.07	0/0.05	1/4.40
3/2.3	2/1.46	0/0.73	0/0.41	0/0.05	0/0.04	5/5
1/1.74	2/0.74	0/0.21	0/0.04	0/0.01	-/-	3/2.74
6/6.8	6/3.91	3/1.47	2/0.37	0/0.1	-/-	17/12.65
15/18.1	10/10.1	2/3.71	0/0.92	1/0.25	-/-	28/33.06

C

GoF = 4.99

Year gap	1	2	3	4	5	6	Total
Parent-offspring							
Maternal	0/1.42	0/1.37	1/0.91	0/0.62	0/0.1	0/0.09	1/4.5
Paternal	3/2.05	2/1.45	0/0.82	0/0.52	0/0.08	0/0.07	5/5
Siblings							
Full	1/1.56	2/0.78	0/0.26	0/0.06	0/0.01	-/-	3/2.66
Maternal half	6/6.25	6/4.06	3/1.72	2/0.51	0/0.15	-/-	17/12.69
Paternal half	15/17.8	10/10.1	2/3.81	0/0.99	1/0.27	-/-	28/32.99

D

GoF = 5.06

1	2	3	4	5	6	Total
0/1.62	0/1.37	1/0.78	0/0.46	0/0.06	0/0.05	1/4.34
3/2.35	2/1.46	0/0.71	0/0.39	0/0.05	0/0.04	5/5
1/1.7	2/0.74	0/0.22	0/0.04	0/0.01	-/-	3/2.7
6/6.96	6/3.87	3/1.41	2/0.35	0/0.09	-/-	17/12.68
15/17.7	10/10.2	2/3.93	0/1.02	1/0.29	-/-	28/33.14

Table S7 Summary of observed versus expected kin. Year gap corresponds to the difference between the juvenile's birth year and the adult's capture year for POPs and the two birth cohort years for SPs. Each cell shows the observed versus (/) the expected number of kin, computed as the number of combinations × kinship probability computed under the maximum likelihood estimates of $\boldsymbol{\theta}$. The sibling rows are differentiated by sex for half-sibling pairs by taking individuals with the same mtDNA haplotype as maternal. The tables are ordered in correspondence with the case table presented in the text i.e., A) (0,0), B) (0,1), C) (1,0) and D) (1,1), for the tuple ($\delta_{♂*}$, $\delta_{♀,♂}$ prior) with 0 representing not included in the model and 1 included. GoF corresponds to the $\sum_k(\text{Obs}_k - \text{Exp}_k)^2/\text{Exp}_k$ measure, where k is the kinship categories and the observed and expected values are taken from the 'Total' column.

A

			95% CI		95% PL-CI	
Parameter	Estimate	SE	Lower	Upper	Lower	Upper
$N_{♀,t_0}$	2,459	1,478	757	7,987	788	8,390
$N_{♂,t_0}$	4,601	3,527	1,024	20,669	1,100	22,500
$N_{♀,t_{2017}}$	2,049	785	967	4,340	991	4,470
$N_{♂,t_{2017}}$	3,834	2,048	1,346	10,921	1,460	12,246
$N_{t_{2017}}$	5,883	2,468	2,585	13,385	2,740	14,820
$\delta_{♀,♂}$	0.198	0.159	0.041	0.952	–	0.523
π	0.230	0.115	0.077	0.515	0.077	0.562
$\varrho_{♀,♂}$	-0.046	0.148	-0.335	0.244	-0.337	0.243
α	5.435	0.563	4.332	6.538	4.065	6.358

B

			95% CI		95% PL-CI	
Parameter	Estimate	SE	Lower	Upper	Lower	Upper
$N_{♀,t_0}$	2,393	1447	732	7,825	762	8,218
$N_{♂,t_0}$	4,505	3,461	999	20,308	1,070	22,075
$N_{♀,t_{2017}}$	1,733	586	893	3,363	920	3,490
$N_{♂,t_{2017}}$	3,263	1,646	1,214	8,769	1,328	9,935
$N_{t_{2017}}$	4,996	1,902	2,368	10,538	2,739	14,820
$\delta_{♀,♂}$	**0.291**	**0.129**	**0.123**	**0.692**	**0.044**	**0.549**
π	0.229	0.114	0.077	0.512	0.076	0.558
$\varrho_{♀,♂}$	-0.081	0.143	-0.361	0.200	-0.363	0.199
α	5.444	0.561	4.345	6.544	4.077	6.363

C

			95% CI		95% PL-CI	
Parameter	Estimate	SE	Lower	Upper	Lower	Upper
$N_{♀,t_0}$	2,635	1,690	750	9,261	780	9,762
$N_{♂,t_0}$	4,982	4,029	1,021	24,315	1,092	26,409
$N_{♀,t_{2016}}$	2,329	1,055	959	5,659	904	4,936
$N_{♂,t_{2016}}$	4,403	2,730	1,306	14,843	1,341	14,872
$N_{t_{2016}}$	6,732	3,442	2,472	18,338	2,464	17,928
$\delta_{♀,♂}$	0.149	0.219	0.008	2.660	–	0.624
$\delta_{♂*}$	**0.244**	**0.218**	**0.042**	**1.402**	–	0.704
π	0.194	0.143	0.039	0.590	0.043	0.791
$\varrho_{♀,♂}$	-0.041	0.148	-0.332	0.249	-0.334	0.249
α	5.448	0.563	4.343	6.552	4.076	6.371

D

			95% CI		95% PL-CI	
Parameter	Estimate	SE	Lower	Upper	Lower	Upper
$N_{♀,t_0}$	2,299	1,442	673	7,857	701	8,270
$N_{♂,t_0}$	4,291	3,409	904	20,360	969	22,100
$N_{♀,t_{2017}}$	1,677	612	821	3,429	847	3,570
$N_{♂,t_{2017}}$	3,131	1,669	1,101	8,900	1,200	10,000
$N_{t_{2017}}$	4,808	1,983	2,143	10,789	2,460	17,900
$\delta_{♀,♂}$	**0.312**	**0.157**	**0.116**	**0.839**	**0.013**	**0.631**
$\delta_{♂*}$	**0.250**	**0.218**	**0.045**	**1.377**	–	**0.711**
π	0.255	0.174	0.054	0.672	0.062	0.924
$\varrho_{♀,♂}$	-0.079	0.143	-0.360	0.202	-0.362	0.201
α	5.435	0.563	4.332	6.539	4.060	6.360

Table S8 Summary of CKMR model parameter estimates and uncertainty across four cases. Hessian-based standard errors (SE) are reported from TMB with 95% Hessian confidence intervals using $z_{1-0.05/2}$ as the SE multiplier constructed and then transformed for those parameters estimated on the log or logit scale. Profile likelihood 95% confidence intervals are also provided for comparison with dashed entries indicating no result from this method. The tables are ordered in correspondence with the case table presented in the text i.e., A) (0,0), B) (0,1), C) (1,0) and D) (1,1), for the tuple ($\delta_{♂*}$, $\delta_{♀,♂}$ prior) with 0 representing not included in the model and 1 included. The parameters $N_{♀,t_0}$and $N_{♂,t_0}$ are the abundance estimate in the first year of modelling (2013) for females and males respectively. Estimates of $N_{♀,t_{2017}}$, $N_{♂,t_{2017}}$ and $N_{t_{2017}}$ (or $_{t_{2016}}$) correspond to the female, male and total adult abundance in the lowest coefficient of variation year. Parameters $\varrho_{♀,♂}$ and $\delta_{♀,♂}$ correspond to the adult trend and mortality parameters estimated together for females and males. Quantities α and π model mate persistence and the proportion of the total adult male abundance contributing to reproduction. The parameter $\delta_{♂*}$ models the mortality of the breeding males. Rows in bold correspond to the different parameters in each model compared to the base model (A).

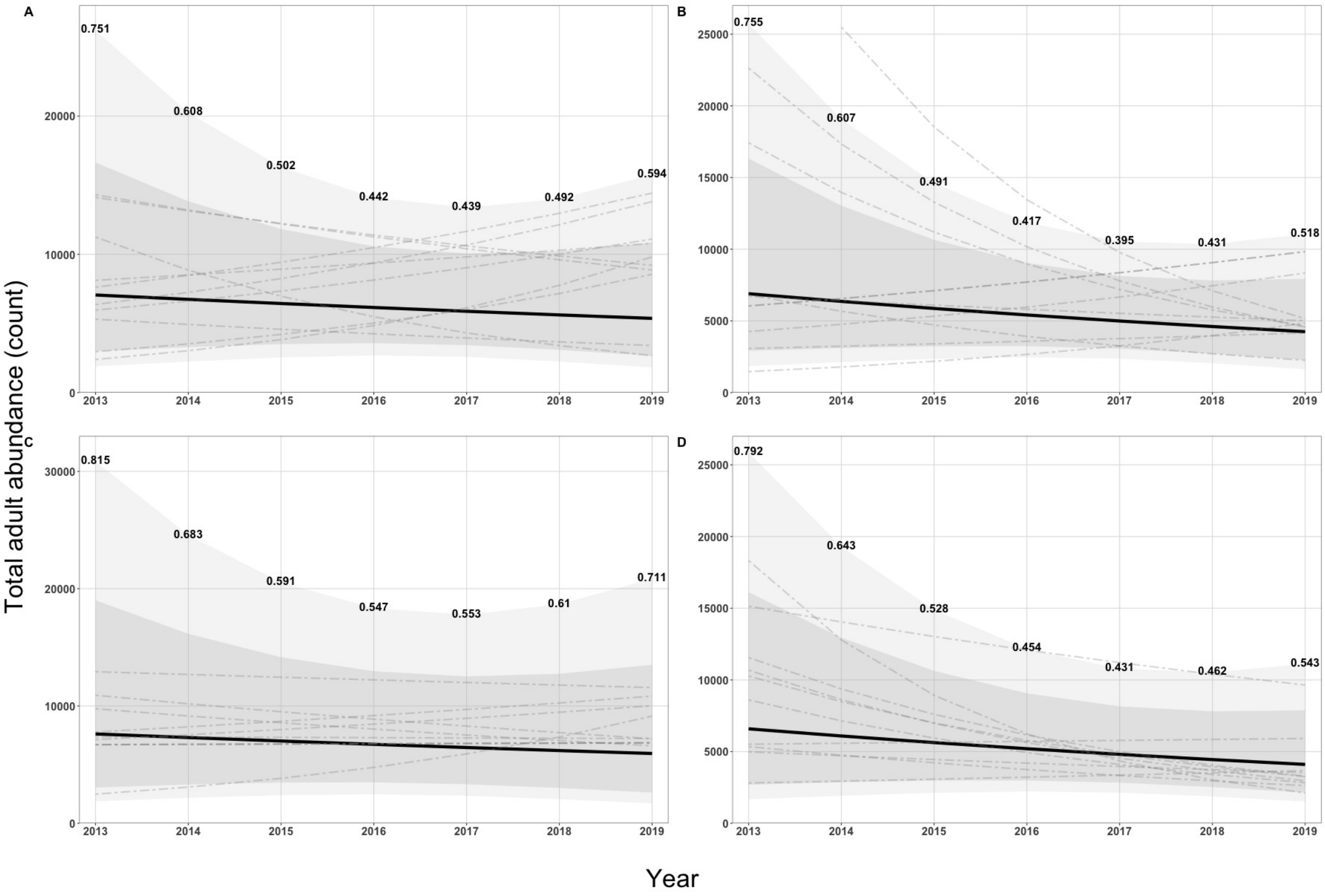

Figure S10 Estimated trend in total adult abundance. The time series of total adult abundance estimated by the CKMR method is shown as a dark solid line. Confidence intervals are depicted with grey shaded areas with 95% external and 80% internal. The coefficient of variation for each year's abundance estimate is reported as bold values above each upper interval estimate. Ten sampling importance resampled *worms* are added to show variability in trends generated from parameter estimates and their variability. The panel order corresponds to the two-by-two table detail in the text of this note. Panel (A) shows the results for the base model.

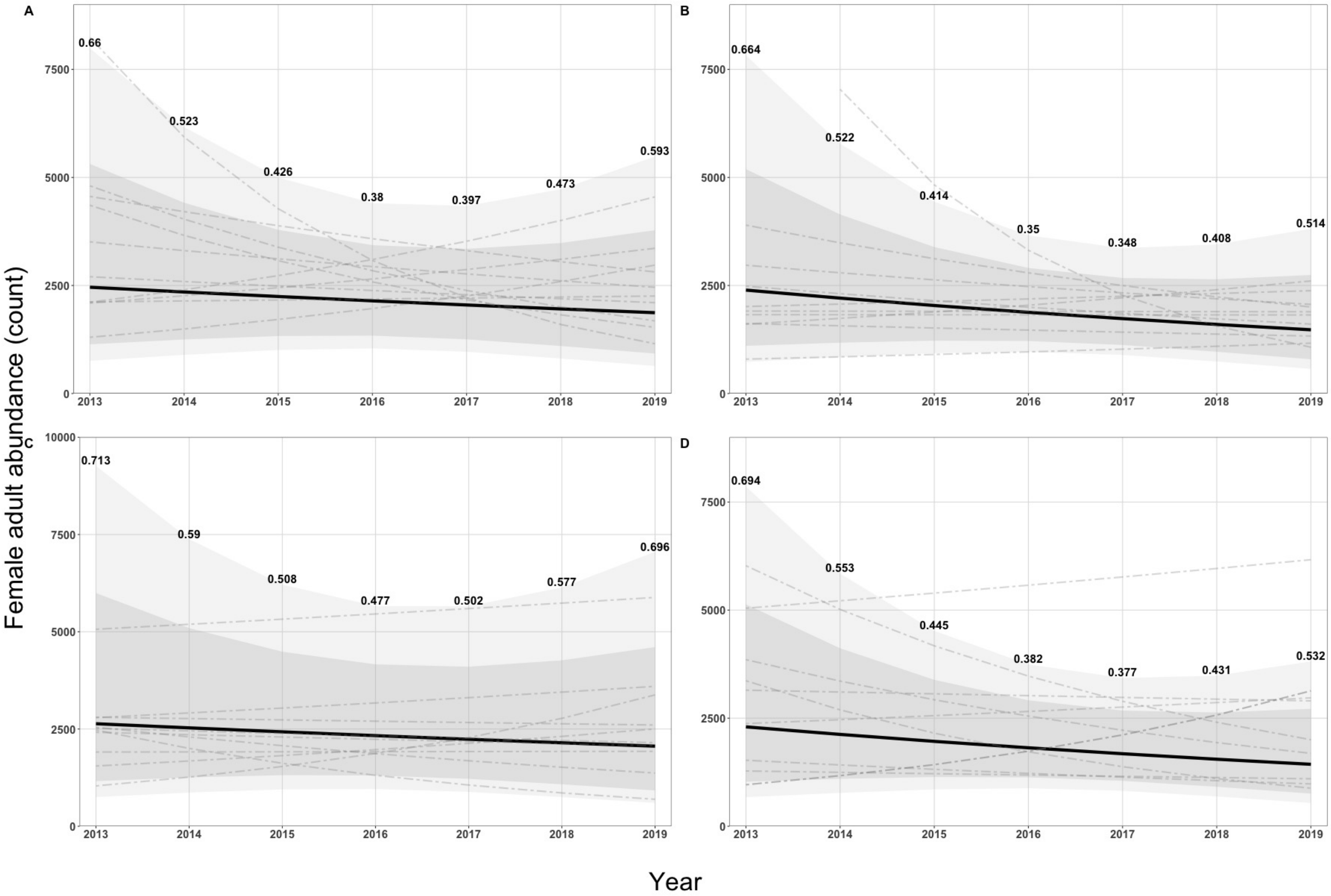

Figure S11 Estimated trend in total female abundance. Time series of total female abundance estimated by CKMR method shown as a dark solid line. Confidence intervals are depicted with grey shaded areas with 95% external and 80% internal. The coefficient of variation for each year's abundance estimate is reported as bold values above each upper interval estimate. Ten sampling importance resampled *worms* are added to show variability in trends generated from parameter estimates and their variability. The panel order corresponds to the two-by-two table detail in the text of this note. Panel (A) shows the results for the base model.

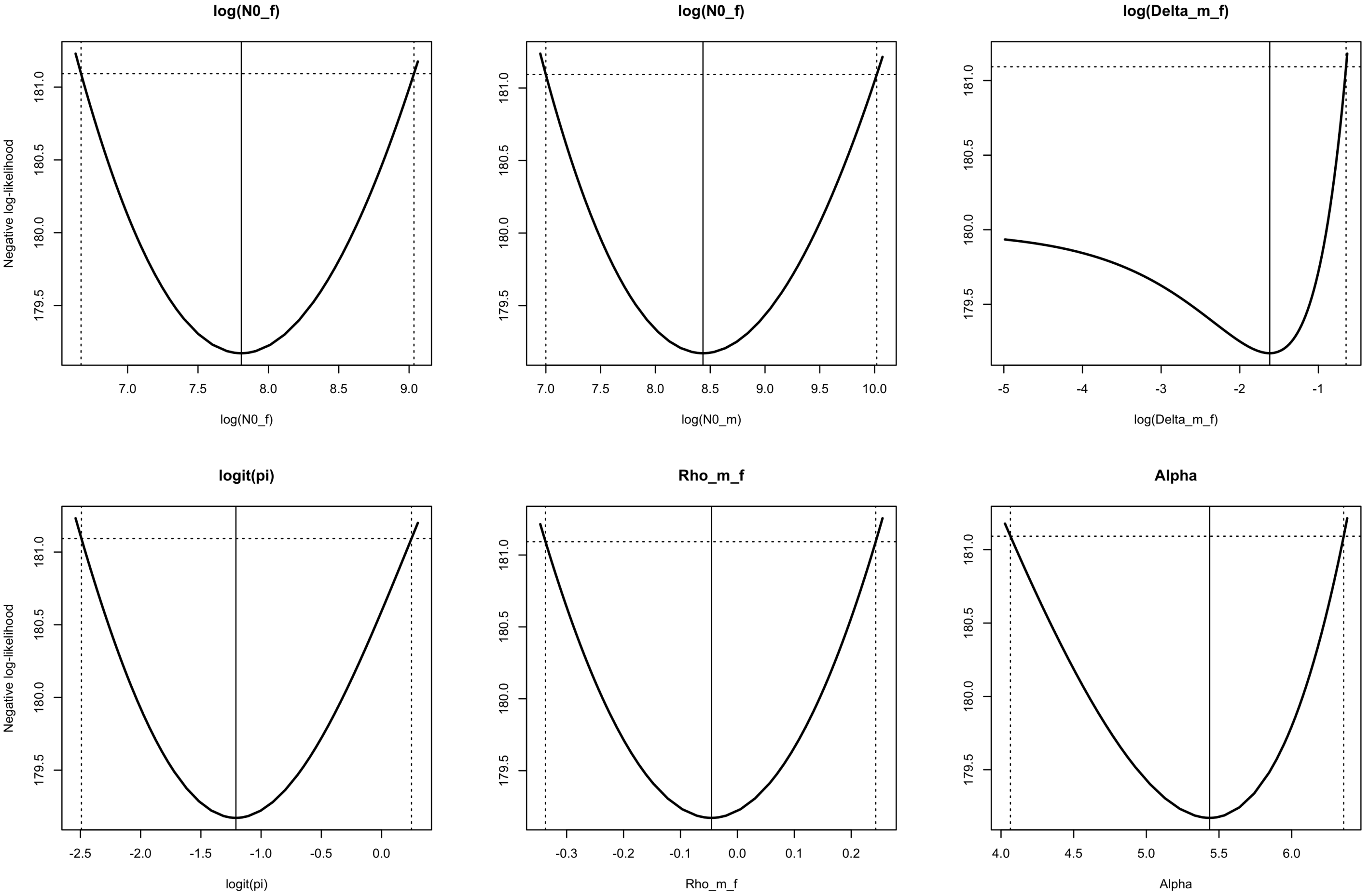

Figure S12 Profile likelihood plots for estimated parameters of base extended model. The x-axes show the range of the parameter on the scale used for model fitting and the y-axes the negative log-likelihood. Labels above each subfigure describe the parameters; from left to right and top to bottom the parameters are $N_{♀,t_0}$ and $N_{♂,t_0}$ on log scale, $\delta_{♀,♂}$ on log scale, $\text{logit}(\pi)$, $\varrho_{♀,♂}$ and α. The left-hand-side boundary of the mortality parameter corresponds to a survival parameter approaching 1 (survival = $\exp(-\exp(\log(\delta_{♀,♂}))$), which implies that CKMR has little information whether individuals survive for long periods of time.

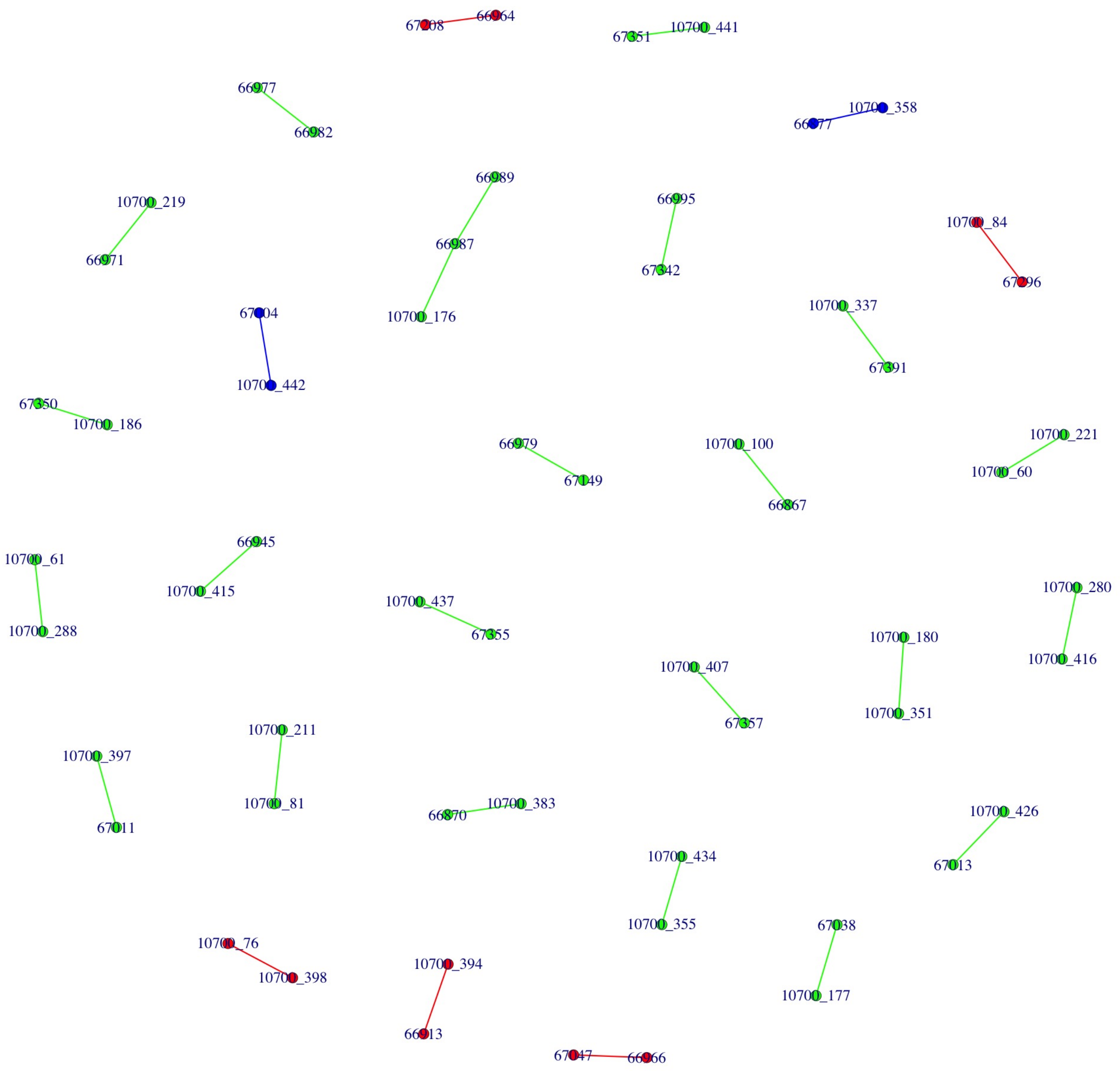

Figure S13 Graph of kin pairs found with same mitochondrial-DNA haplotypes. Each node is an individual and the link between individuals implies they are a kin pair. The colour of the link and node informs the kinship relationship determined with green for HSPs, red for FSPs and blue for mother-offspring pairs. All pairs found are shown and may differ from the number reported in the main text as some pairs were removed based on ageing criteria.

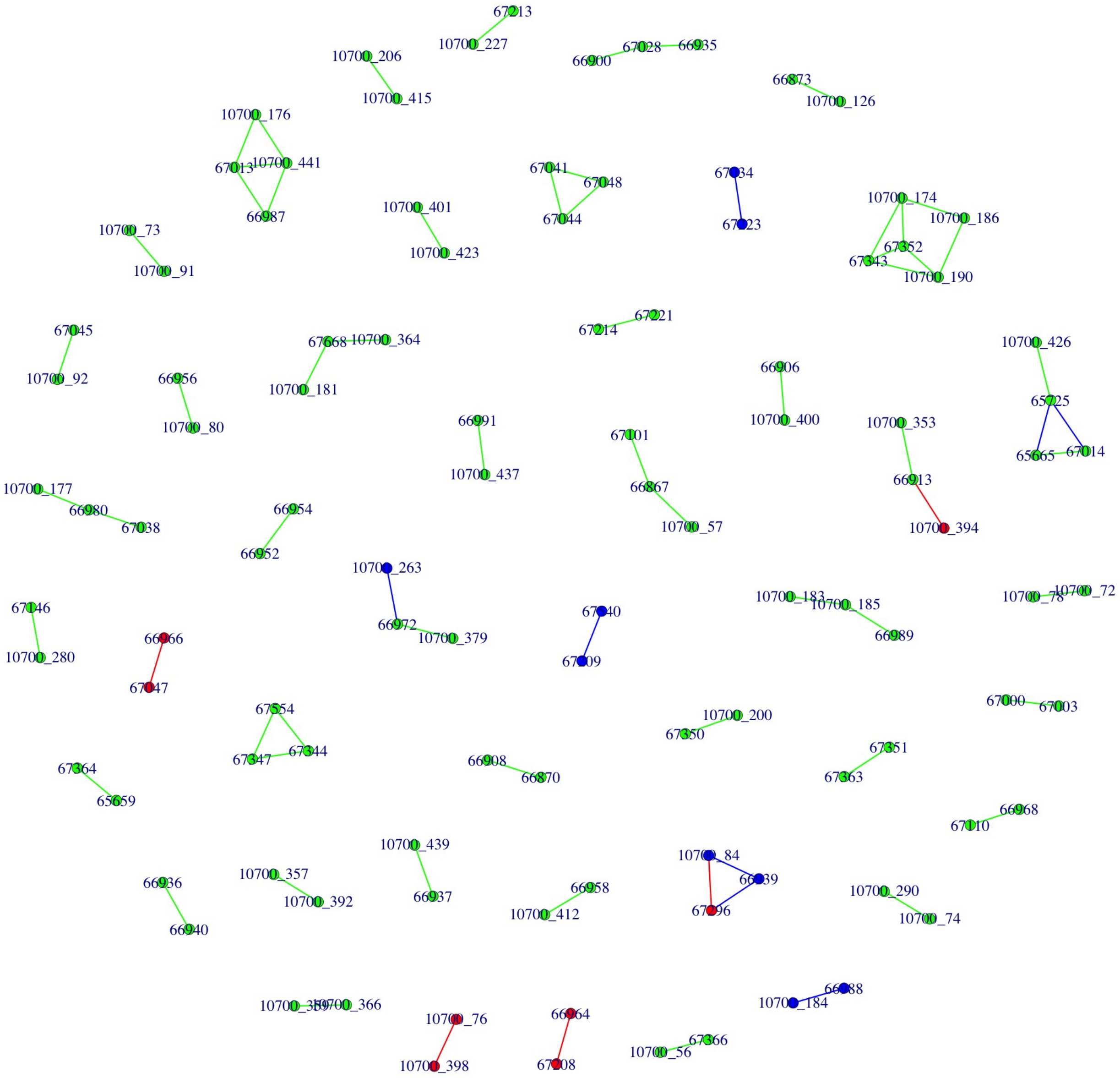

Figure S14 Graph of kin pairs found with different mitochondrial-DNA haplotypes. Each node is an individual and the link between individuals implies they are a kin pair. The colour of the link and node informs the kinship relationship determined with green for half-sibling pairs, red for full-sibling pairs and blue for father-offspring pairs. All pairs found are shown and may differ from the number reported in the main text as some pairs were removed based on ageing criteria.

References

[1] Paradis, E. pegas: an R package for population genetics with an integrated–modular approach. *Bioinformatics* **26**, 419–420 (2010).

[2] Sansaloni, C. *et al.* Diversity Arrays Technology (DArT) and next-generation sequencing combined: genome-wide, high throughput, highly informative genotyping for molecular breeding of Eucalyptus. In *BMC proceedings*, vol. 5, P54 (BioMed Central, 2011).

[3] Kilian, A. *et al.* Diversity arrays technology: a generic genome profiling technology on open platforms. In *Data production and analysis in population genomics*, 67–89 (Springer, 2012).

[4] Melville, J. *et al.* Identifying hybridization and admixture using snps: application of the dartseq platform in phylogeographic research on vertebrates. *Royal Society open science* **4**, 161061 (2017).

[5] Bravington, M. V., Miller, D. L. & Baylis, S. M. *Kinference: pair wise kin-finding for close-kin mark-recapture* (2019). R package version 0.0.80.

[6] Hillary, R. *et al.* Genetic relatedness reveals total population size of white sharks in eastern Australia and New Zealand. *Scientific Reports* **8**, 1–9 (2018).

[7] Bravington, M. V., Skaug, H. J., Anderson, E. C. *et al.* Close-kin mark-recapture. *Statistical Science* **31**, 259–274 (2016).

[8] Todd, C. M. The ecology and conservation of the Christmas Island flying-fox (Pteropus natalis). *PhD Thesis - Western Sydney University* (2020).

[9] Phalen, D. N. *et al.* Genetic diversity and phylogeny of the Christmas Island flying fox (Pteropus melanotus natalis). *Journal of Mammalogy* **98**, 428–437 (2017).

[10] O'Brien, J. *et al.* Multiple colonisations of the western indian ocean by pteropus fruit bats (megachiroptera: Pteropodidae): the furthest islands were colonised first. *Molecular Phylogenetics and Evolution* **51**, 294–303 (2009).

[11] Hall, T. BioEdit: a user-friendly biological sequence alignment editor and analysis program for Windows 95/98NT **41**, 95–98 (1999).
